# Predicting chemotherapy responsiveness in gastric cancer through machine learning analysis of genome, immune, and neutrophil signatures

**DOI:** 10.1007/s10120-024-01569-4

**Published:** 2024-12-02

**Authors:** Shota Sasagawa, Yoshitaka Honma, Xinxin Peng, Kazuhiro Maejima, Koji Nagaoka, Yukari Kobayashi, Ayako Oosawa, Todd A. Johnson, Yuki Okawa, Han Liang, Kazuhiro Kakimi, Yasuhide Yamada, Hidewaki Nakagawa

**Affiliations:** 1 Laboratory for Cancer Genomics, RIKEN Center for Integrative Medical Sciences, Yokohama, 230-0045 Japan; 2 Department of Head and Neck, Esophageal Medical Oncology, National Cancer Center Hospital, Tokyo, Japan; 3 Precision Scientific (Beijing) Ltd, Beijing, 100085 China; 4 Department of Immunotherapeutics, The University of Tokyo Hospital, Bunkyo-ku, Tokyo, 113-8655 Japan; 5 Department of Immunology, Faculty of Medicine, Kindai University, Sayama, Osaka 589-8511 Japan; 6 Department of Bioinformatics and Computational Biology, Department of Systems Biology, The University of Texas MD Anderson Cancer Center, Houston, TX 77030 USA; 7 Department of Medical Research, National Center for Global Health and Medicine, Tokyo, 162-8655 Japan

**Keywords:** Gastric cancer, Chemotherapy, Whole-genome sequencing., RNA sequencing, Machine learning, Tumor-associated neutrophils, Personalized medicine

## Abstract

**Background:**

Gastric cancer is a major oncological challenge, ranking highly among causes of cancer-related mortality worldwide. This study was initiated to address the variability in patient responses to combination chemotherapy, highlighting the need for personalized treatment strategies based on genomic data.

**Methods:**

We analyzed whole-genome and RNA sequences from biopsy specimens of 65 advanced gastric cancer patients before their chemotherapy treatment. Using machine learning techniques, we developed a model with 123 omics features, such as immune signatures and copy number variations, to predict their chemotherapy outcomes.

**Results:**

The model demonstrated a prediction accuracy of 70–80% in forecasting chemotherapy responses in both test and validation cohorts. Notably, tumor-associated neutrophils emerged as significant predictors of treatment efficacy. Further single-cell analyses from cancer tissues revealed different neutrophil subgroups with potential antitumor activities suggesting their usefulness as biomarkers for treatment decisions.

**Conclusions:**

This study confirms the utility of machine learning in advancing personalized medicine for gastric cancer by identifying tumor-associated neutrophils and their subgroups as key indicators of chemotherapy response. These findings could lead to more tailored and effective treatment plans for patients.

**Supplementary Information:**

The online version contains supplementary material available at 10.1007/s10120-024-01569-4.

## Background

Gastric cancer (GC) is a major global health issue, especially prevalent in Japan and Asian countries, where it constitutes a significant public health challenge [1–3]. Despite progress in treatments enhancing survival rates, the recurrence of advanced GC continues to pose a substantial risk [4]. In Japan, the primary treatments for advanced GC involve surgical resection and combination chemotherapy, both of which significantly influence patient outcomes [5]. For those with recurrent or metastatic GC, the standard care includes chemotherapy alongside an immune checkpoint inhibitor for HER2-negative cases, trastuzumab for HER2-positive cases, and zolbetuximab for Claudin 18.2-positive cases [6] [7] [8]. Furthermore, perioperative chemotherapy, based on fluoropyrimidines and platinum, has become widely adopted for locally advanced GC, underscoring the importance of identifying suitable candidates for this treatment to enhance patient survival [9] [10].

GC’s molecular classification identifies four primary subtypes, each distinguished by specific genetic and molecular features. These include the Epstein–Barr virus (EBV)-positive type, characterized by EBV infection; the microsatellite instability-high (MSI-high) type, known for its robust response to immune checkpoint inhibitors due to DNA mismatch repair defects; the chromosomal instability (CIN) type, noted for chromosomal instability and *TP53* mutations; and the genomically stable (GS) type, with fewer genomic alterations but notable mutations. These classifications are instrumental in tailoring personalized treatment approaches [11], particularly for HER2-positive GC, predominantly found in the CIN subtype and treated with trastuzumab. However, these molecular subtypes have not been directly linked to chemotherapy responses, except in the context of HER2-targeted therapy.

The variability in chemotherapy effectiveness among patients highlights the complexity of predicting treatment outcomes. In this scenario, machine learning/AI technologies emerge as a promising solution. By integrating clinical and molecular biological factors through machine learning or AI algorithms, we can more accurately forecast chemotherapy results [12] [13] [14]. This advancement paves the way for more personalized, patient-specific treatment plans. Moreover, the tumor’s immune environment, including the role of immune cells such as neutrophils in cancer proliferation and metastasis, is fundamental to understanding GC’s growth and progression [15]. Exploring how these cells interact with chemotherapy may unlock more effective treatment modalities [16].

Our study aims to create a predictive model for chemotherapy effectiveness using the machine learning method, incorporating molecular profiles and immune signatures. This model strives to facilitate personalized treatment options for GC patients, ultimately enhancing survival rates.

## Materials and methods

### Experimental design

The primary objective of this study was to develop a predictive model capable of accurately determining chemotherapy responsiveness in GC patients. This was achieved through a translational study conducted at the National Cancer Center Hospital in Japan, where subjects with pathologically confirmed advanced GC were enrolled based on specific inclusion criteria. These criteria included having a primary lesion and at least one target lesion, planned HER2 testing and palliative chemotherapy, and being 20 years of age or older. Our design incorporated the collection and analysis of clinical data, genomic information, immune signatures, and copy number signatures from these patients. Tissue samples were collected at predetermined points before and after palliative chemotherapy and at tumor progression, ensuring a comprehensive dataset for model development. The study’s design was pre-specified to include these components, ensuring a structured approach to achieving our research goals.

### Experimental model and subject details

All subjects were enrolled in the translational study conducted at the National Cancer Center Hospital. Inclusion criteria were as follows: (i) pathologically confirmed advanced GC, (ii) had a primary lesion and at least one target lesion, (iii) HER2 test and palliative chemotherapy were planned, and (iv) 20-year-old or more. Informed consent was obtained from all participants who were registered from January 2013 to December 2017. The study was approved by the institutional review boards of the National Cancer Center (2012–118) and is registered with the University Hospital Medical Information Network Clinical Trials Registry (UMIN-CTR), number 000009564.

### Tissue sample characteristics

In the translational study, the sampling of blood and the fresh core biopsy was performed at three points: (i) before palliative chemotherapy, (ii) after two courses of palliative chemotherapy, and (iii) just after tumor progression. Tumor samples were obtained from a primary lesion using upper gastrointestinal endoscopy.

### Bulk RNA sequencing

RNA was extracted from frozen biopsy specimens collected before chemotherapy using TRIzol reagent. The quality and quantity of total RNA were assessed using a Bioanalyzer (Agilent Technologies). RNA sequencing libraries were generated using the TruSeq Stranded mRNA Library Preparation Kit (Illumina), and sequencing was performed on a HiSeq2500 (Illumina). Read mapping to the human reference genome (GRCh38) was conducted using TopHat2 (https://ccb.jhu.edu/software/tophat/index.shtml), and per-gene read counting was performed using GENCODE release 19 with HTseq, all orchestrated by the iRAP pipeline (https://github.com/nunofonseca/irap). The gene expression levels were quantified as fragments per kilobase of exon per million fragments mapped with upper quartile normalization (FPKM-UQ). For Z-score calculations, the expression values were log-transformed by adding an offset of 0.01 to FPKM-UQ and then taking the natural logarithm (log(1 + FPKM-UQ)). The log-transformed data were then scaled across rows (genes) to center the mean expression at zero and standardize the variance, resulting in Z-scores. Specifically, the data matrix was transposed, scaled using the scale function, and then transposed back. These Z-scores were subsequently used in the heatmap visualization to highlight relative expression changes across samples.

### Differential expression gene and pathway analysis

Differential gene expression analysis was conducted using the edgeR [17] and limma [18] packages in R. Briefly, count data were normalized using the trimmed mean of M values method. The differences in gene expression were evaluated using the voom function of the limma package, linear modeling, and empirical Bayes moderation. Differentially expressed genes were defined as those with an adjusted P-value < 0.05 and |log2 fold change|> 1, where P-values were adjusted for multiple testing using the False Discovery Rate (FDR) method. Gene Set Enrichment Analysis (GSEA) [19] was performed on genes sorted by the t-statistics calculated in the differential gene expression analysis, using the clusterProfiler package in R [20]. In this analysis, P-values were adjusted using the Benjamini and Hochberg method, with a cutoff value set at 0.05. GSEA was carried out using the Hallmark gene sets (H) and ontology gene sets (C5) downloaded from the Molecular Signatures Database.

### Single-cell RNA sequencing (scRNA-seq) of frozen tumor digests (FTDs)

Fresh tumor samples were sectioned into small fragments and subjected to enzymatic dissociation using a tumor dissociation kit (Miltenyi Biotec Inc., Auburn, CA, USA), following the manufacturer’s guidelines to produce fresh tumor digests (FTDs). These FTDs were then strained through a 70-μm cell strainer (Thermo Fisher Scientific, Hampton, NH, USA) and cryopreserved in Bambanker™ freezing medium (NIPPON Genetics, Tokyo, Japan) for subsequent analysis. For the analysis, the cryopreserved FTDs were thawed in RPMI medium and stained with Fixable Viability Dye eFluor™780 (Thermo Fisher Scientific) along with PacificBlue-labeled anti-human CD45 antibody (clone HI30, BioLegend, San Diego). The live CD45 + cells were isolated using a Cell Sorter SH800S (SONY, Tokyo, Japan). These sorted cells were resuspended at a concentration of 1000 cells/μl in 0.04% BSA/PBS, and then loaded onto a Chromium Controller (10xGenomics, Pleasanton, California, USA) for processing. cDNA libraries were prepared using the Chromium Single Cell 5′ Reagent Kits v2 (10xGenomics) as per the manufacturer’s instructions. The prepared library was finally sequenced using the HiSeq2500 system (Illumina).

### Raw sc-RNAseq read alignment quality control and normalization.

The Cell Ranger Single Cell Software Suite v.6.1.2 (https://www.10xgenomics.com/jp/support/software/cell-ranger/latest) was employed to process, align, and summarize unique molecular identifier (UMI) counts following the standard pipeline and default parameters. For quality control in Seurat (https://satijalab.org/seurat/) analysis, the raw UMI count matrix was filtered to remove genes expressed in fewer than three cells and cells expressing fewer than 100 genes or having more than 50% mitochondrial gene counts. The potential doublets in each sample were identified using the R package scDblFinder (v1.6.0) with the parameter dbr.sd = 1 [21]. The resulting matrix was normalized using the global-scaling method, transformed with a scaling factor (default of 10,000 by), and log-transformed with Seurat's "LogNormalize" function for downstream analysis. For assigning individual cell types, the Monaco immune data was used as a reference dataset with the R package SingleR [22] employing default parameters. Each single cell was annotated with a cell type in the dataset’s label.main.

### Identification of neutrophil subclusters

Neutrophil fractions labeled using SingleR (https://github.com/dviraran/SingleR) were extracted for further clustering analysis, excluding clusters and cells with high frequencies of immunoglobulins or those expressing suspicious lineage marker genes such as *CD3E, CD19, NCAM1, CD8A*, and *CD4*. To identify the characteristics of neutrophil subclusters, single-cell RNAseq data of neutrophils obtained from the previous study by Roudong Xue et al. [23] and our own neutrophil single-cell RNAseq data were compared using Cosine similarity. Furthermore, to define the features of the fractions, marker genes for TAN1 (*CXCL8, CXCL1, CXCL2, ICAM1, CD44*), TAN2 (*HLA-DRA, CD74, HLA-DPB1*), TAN3 (*PLIN2, PLAU*), TAN4 (*RPL10, PRS2, RPS18, RPL3*), and NETosis (*PROK2, MME*) [24] [25] were used to assess expression in each cluster. The signatures of neutrophils were calculated by taking the square root of the product of the top 10 genes specifically expressed in the cell fractions of anti-tumor neutrophils and pro-tumor neutrophils, and these were used as respective signatures. Single-cell RNAseq plots and analyses were conducted using the SCP (https://github.com/zhanghao-njmu/SCP) and SCPubr (https://enblacar.github.io/SCpubr-book/) packages.

### Spatial transcriptome (ST) analysis

Spatial transcriptome data of human gastric mucosa [26] were obtained from Zenodo (https://doi.org/10.5281/zenodo.8333281). The data were downloaded and processed using Seurat, following the methods described in their paper.

### Whole genome sequencing and copy number analyses

DNA extraction from both frozen tumor and normal mucosa biopsy specimens was performed using the QIAamp DNA Mini Kit (QIAGEN). Library preparation followed the manufacturer’s instructions, utilizing the TruSeq Nano DNA Library Prep Kit (Illumina). Paired-end sequencing with 125-bp reads was executed on the HiSeq2500 system. In our analysis of 65 matched tumor and normal pairs, shallow whole-genome sequencing (sWGS) was employed at an approximate depth of 1.0 × for both tumor and normal samples. The sequence reads were aligned to the human reference genome GRCh38 using BWA-0.7.8 (https://github.com/lh3/bwa). Subsequently, PCR duplicates were removed using the Picard toolkit. For the identification of copy number amplified and deleted regions, segment files generated from the QDNAseq [27] and ABSOLUTE [28] pipeline were analyzed. The segment files generated from the QDNAseq pipeline were used to calculate copy number amplified and deleted regions, using the default settings of the GISTIC2 (v7). Using Sigminer [29], we calculated copy number signatures from segment files based on COSMIC. We then assigned the characteristics of COSMIC signatures [30] to our GC copy number signatures using Cosine similarity. These bioinformatics tools using the default settings.

### Target deep sequencing and variant detection

To detect and validate low-VAF (variant allele frequency) mutations in GC, RNA capture probes (SureSelect) were designed to include the coding regions of previously reported GC-related genes (Supplementary Table 1). Sequencing libraries were constructed from 50 ng of DNA input using SureSelect XT low input reagents, with molecular barcoding and target capturing performed by the SureSelect XT Target Enrichment System (Agilent). The target-captured libraries were sequenced on the HiSeq2500 with paired 125-bp reads. After adjusting molecular barcodes and removing duplication, the average depth of the target regions was 1500x. Somatic mutations were detected and filtered using Mutect2. The specimens without mutations in any of the three MSI markers, BAT25, MS05, and MS11, are considered to have microsatellite stability (MSS). Those with mutations in any one of these genes are categorized as having MSI-low, and those with mutations in two or more of these genes are classified as having MSI-high. Each SNV was drawn using maftools [31].

### Decision tree and prediction model

The integrated machine learning analysis of our data utilized the rpart (https://github.com/bethatkinson/rpart) and Random Forest [32] R packages. We divided our cohort data randomly into a training set (70%) and a testing set (30%). This 70/30 split ratio is a standard practice for mid-sized samples in machine learning applications, selected to balance the need for ample training samples to build a robust model and sufficient test samples for model evaluation. Additionally, we prepared validation data consisting of GC patients from China (*N* = 35) [33]. To identify key factors from the 123 elements, we employed the Boruta package. The partition tree (RPART) model is a classification approach based on a top-down methodology, initiating from a root node and generating binary splits in the data until a specific criterion, such as the minimization of node impurity, is met. This method can tend to overfit the training data. The cross-validation or bootstrapping procedures are valuable in limiting overfitting, leading to the proper tuning of decision tree (DT) parameters and optimizing model accuracy. The Random Forest algorithm, a tree-based method, involves computing hundreds to thousands of RPART trees and combining the DT outputs, enhancing the model's generalizability. The ROCR R package was applied for AUC evaluation of the response classification accuracy based on out-of-bag predictions. To visualize the decision tree from the diagnostic model, multiple trees from the random forest were distilled into a single representative tree. In the distillation process, a method that considers the balance between predictive accuracy and interpretability was employed. Specifically, a tree containing the most important features was selected from the multiple trees, and a single decision tree was constructed based on it.

### Statistical analyses

In our study, statistical analyses were conducted with meticulous attention to detail to ensure the robustness and reliability of our findings. The primary method for comparing the chemotherapy response between responder and non-responder groups was the two-sided Mann–Whitney U-test, chosen for its non-parametric nature, allowing for comparisons without assuming a normal distribution of the continuous variables under investigation. To assess the strength and direction of the association between two continuous variables, we utilized Spearman’s rank correlation coefficient, providing insight into relationships that are not necessarily linear. Furthermore, to predict the response to chemotherapy more comprehensively, we employed logistic regression modeling. This approach was utilized in both univariate and multivariate formats, enabling us to identify individual predictors of chemotherapy response as well as how they interact when considered simultaneously. The logistic regression analyses were crucial for developing a predictive model that could potentially guide personalized treatment strategies for gastric cancer patients. All statistical analyses were performed using R (version 4.2.1), a software environment for statistical computing and graphics. This choice allowed for sophisticated data analysis and the creation of high-quality graphical representations of our data. To ensure the transparency and replicability of our results, we included specific values for the number of subjects (N), the significance level (P), and the statistical tests performed in the appropriate figure legends or the main text. Such detailed reporting is intended to enable knowledgeable readers with access to the original data to verify our results comprehensively. This rigorous approach to statistical analysis underpins the conclusions drawn from our study and contributes to its potential impact on advancing personalized treatment strategies in gastric cancer. By providing a clear and detailed description of our methods and the statistical analyses employed, we aim to facilitate the replication and verification of our findings by other researchers in the field.

## Results

### Clinical characteristics and chemotherapy *response*

**Fig. 1:**
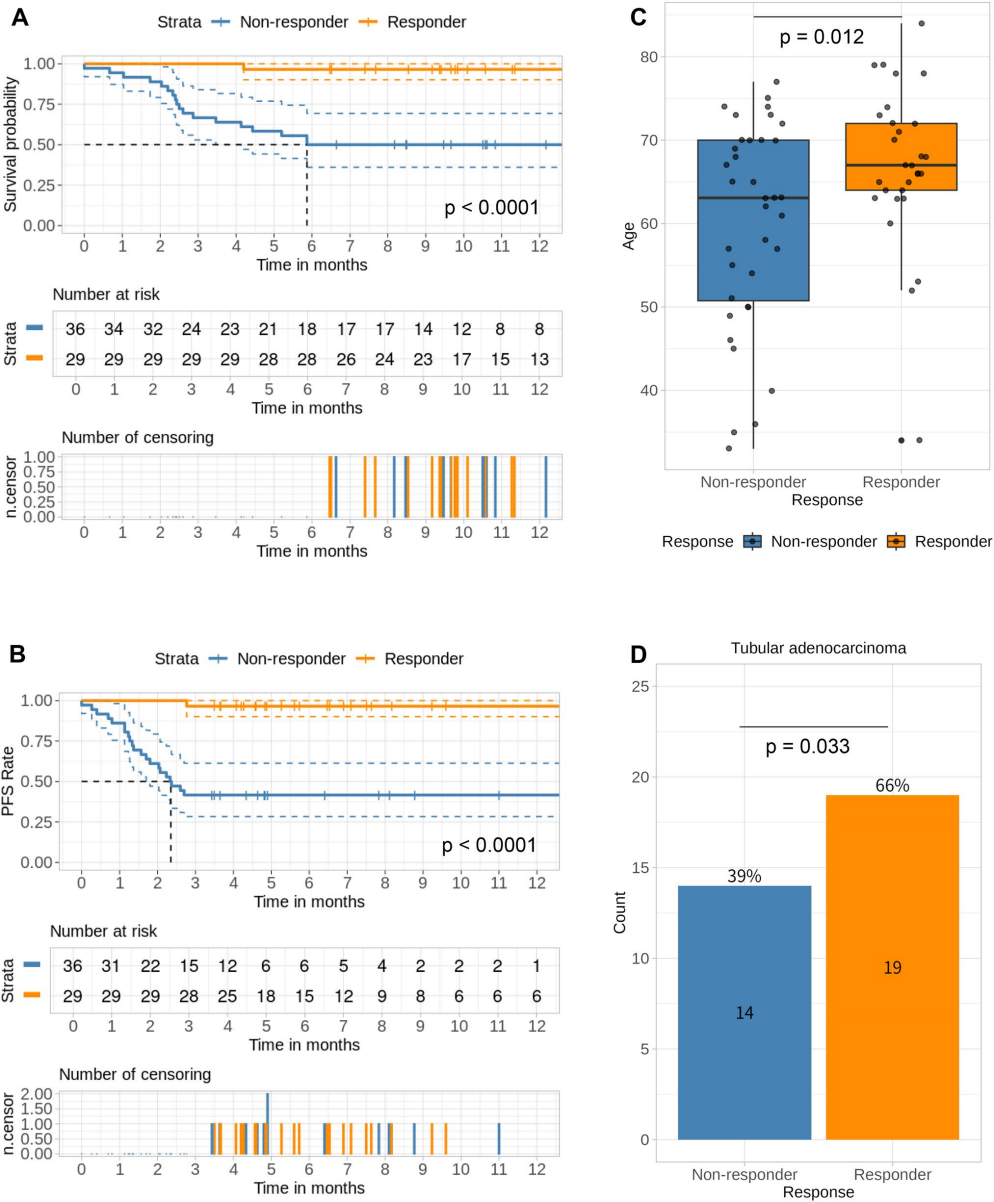
Clinical data from a cohort of 65 GC patients. **A** Overall survival (OS) and **B** Progression-free survival (PFS) in the two groups of chemotherapy Responders and Non-responders. **C** Comparison of age at diagnosis in the two groups of chemotherapy Responders and Non-responders. **D** Incidence of GC tissue type (Tub) in the two groups of chemotherapy Responders and Non-responders

We conducted a comprehensive genomic and transcriptomic analysis of the tumor biopsy specimens before chemotherapy from 65 treatment-naïve unresectable or metastatic GC patients, which were enrolled in a translational study conducted at the National Cancer Center Hospital, Japan, from January 2013 to December 2017. Almost subjects (91%; 59/65) were treated with palliative chemotherapy containing fluoropyrimidine plus platinum agents. The details of the subjects are shown in Table 1. The patients who received chemotherapy were classified into five groups: FP (fluoropyrimidines + platinum), FP + Docetaxel, FP + HER2 (Trastuzumab), PTX (Paclitaxel), and others. Comparing the age (Supplementary Fig. 1A. *P*-value = 0.99 by ANOVA test), gender (Supplementary Fig. 1B. *P*-value = 0.63 by Pearson’s Chi-squared test), and disease status (Supplementary Fig. 1C. *P*-value = 0.86 by Pearson’s Chi-squared test) of patients who received chemotherapy, no significant differences were observed. Therefore, we considered the impact of treatment differences on the outcomes to be negligible. Of the 65 patients, the objective responses were as follows: Complete Response (CR) in 0 cases, Partial Response (PR) in 30 cases, Stable Disease (SD) in 16 cases, and Progressive Disease (PD) in 19 cases. Patients who showed PR were classified as 'Responder,' while those who showed SD or PD were classified as 'Non-responder.' Based on these classifications, we examined its association of clinico-pathological information (Table 1). Differences in response rates among different treatment regimens were also not observed (Supplementary Fig. 1D). The comparison of the 12-month survival period and progression-free survival (PFS) of Responders and Non-responders is shown in Fig. 1A and B. The responders showed better outcomes compared to Non-responders (Fig. 1A: *P* < 0.0001 by log rank test, and Fig. 1B: *P* < 0.0001 by log rank test). Furthermore, between Responders and Non-responders, significant differences were observed in their age (Fig. 1C) and histologic type (Fig. 1D, Supplementary Fig. 2A, and (Table 1), and trends were observed in cT:4a and cN:0 (Supplementary Fig. 2B and 2C, and Table 1).

**Table 1:** GC Patients characteristics

Characteristic	Number of patients(N = 65)		Number of Non-responder(N = 36)	%	Number of Responder(N = 29)	%
Age(median)	33–84(65)		33–77(63)		34–84(67)	
Gender						
Female	20	31	13	36	7	24
Male	45	69	23	64	22	76
Disease status						
Metastatic	63	97	34	94.4	29	100
Unresectable	1	1.5	1	2.8	0	0
NA	1	1.5	1	2.8	0	0
Histology						
Intestinal	44	68	25	69.4	19	66
Diffuse	19	29	9	25	10	34
NEC	2	3	2	5.6	0	0
Tissue type						
Muc	4	6	3	8	1	3
NEC	2	3	2	6	0	0
Por	12	18	9	25	3	10
Sig	14	22	8	22	6	21
Tub	33	51	14	39	19	66
cT						
2	2	3	0	0	2	7
3	9	14	3	8.3	6	21
4a	44	68	28	77.8	16	55
4b	8	12	3	8.3	5	17
NA	2	3	2	5.6	0	0
cN						
0	8	12	7	19	1	3
1	16	25	8	22	8	28
2	16	25	9	25	7	24
3	23	35	10	28	13	45
NA	2	3	2	6	0	0
Liver metastasis						
Yes	21	32	8	22	13	45
No	44	68	28	78	16	55
Peritoneal metastasis						
Yes	32	49	19	53	13	45
No	33	51	17	47	16	55
HER2 status						
Positive	14	22	5	13.8	9	31
Negative	49	75	29	80.6	20	69
NA	2	3	2	5.6	0	0
Chemotherapy						
F + P	31	##	17	47.2	14	48.3
F + P + Docetaxel	17	##	10	27.8	7	24
F + P + Trastuzumab	10	##	3	8.33	7	24
PTX	4	6.2	4	11.1	0	0
Others	3	5	2	5.56	1	3.5

*Muc* Mucinous adenocarcinoma, *NEC* Neuroendocrine carcinoma, *Por* Poorly differentiated adenocarcinoma, *Sig* Signet ring cell carcinoma, *Tub* Tubular adenocarcinoma, *HER2* Human Epidermal Growth Factor Receptor 2, *F* fluoropyrimidines. Most fluoropyrimidine consisted of S-1 or capecitabine, *P* platinum, *PTX* Paclitaxel.

### Genomic and copy number profiles of 65 GCs

DNA and RNA were extracted from fresh frozen core tumor biopsies from GC tissues before chemotherapy, and shallow whole-genome sequencing (× 1 ~ ; 65 cases) and RNA sequencing (65 cases) were performed. In addition, we made a panel of GC-related genes including 67 genes, microsatellite markers, drug-metabolizing genes, EBV and *Helicobacter pylori* (Supplementary Table 1), and targeted deep sequencing (65 cases) was also performed to detect GC-specific variants, pharmacogenomics (PGx) variants, and the pathogens. As a result, we detected 537 variants in the 56 genes (Supplementary Tables 2, 3). As shown in Supplementary Fig. 3A, somatic mutations were frequently found in several driver genes such as *TP53* and *MUC16*; however, no difference in responsiveness to chemotherapy was observed in the presence of these variants (*P* > 0.05 Fisher’s exact test, Supplementary Fig. 3A, and Supplementary Table 1).

Considering the potential relevance of PGx variants to chemotherapy responsiveness due to their influence on individual drug metabolism, drug efficacy, and side effects, we evaluated six genes including *GSTP1, DPYD, CYP2A6, TYMS, DPYS,* and *UGT1A1*. We found that 32/65 (49.2%) of the GC samples had at least one PGx variant in the four genes excluding *TYMS* and *DPYS* (Supplementary Fig. 3B). However, no difference in responsiveness to the FP-based chemotherapy was observed (*P* > 0.05 Fisher’s exact test, Supplementary Fig. 3B, and Supplementary Table 1). Regarding to MSI status of 65 GCs, seven GCs showed MSI-high (Supplementary Fig. 3C), however, no difference in responsiveness to chemotherapy was observed in MSI/MSS (*P* > 0.05 Fisher’s exact test, Supplementary Table 1, and Supplementary Fig. 3C). EBV was detected with a read count ten times higher in three GCs (3/65; 4.6%) compared to other samples. *Helicobacter pylori* was detected in 29/65 (44.6%) of GC tissues, with ten cases showing more than tenfold the number of reads (Supplementary Fig. 3D). However, no difference in responsiveness to chemotherapy was observed in the presence of these pathogens (*P* > 0.05 Fisher’s exact test, Supplementary Table 1, and Supplementary Fig. 3D).

Subsequently, we analyzed somatic copy number alterations (CNAs) of GCs. Using genomic identification of significant targets in cancer (GISTIC) to identify statistically significant CNAs from the whole-genome sequencing data of 65 GCs, we detected copy number changes encompassing genes such as *EGFR, MYC, CCND1, GATA6, CCNE1*, and their surrounding regions, which have been reported to be associated with GC (Supplementary Fig. 4A). But no difference in responsiveness to chemotherapy was observed (*P* > 0.05 by *t*-test, Supplementary Fig. 4B). Next, we calculated the copy number signatures (CNS) for GC, which are patterns of somatic copy number variations (CNVs) that are characteristic of specific cancer types or subtypes. These signatures provide insights into how cancer cells accumulate genomic changes and the mechanical stress and DNA damage responses involved in the process [30], and we evaluated the CNS by using methods in the catalogue of somatic mutations in cancer (COSMIC) [30]. As a result, we extracted two types of CNS (GC_CNSignature1:Sig1 and GC_CNSignature2:Sig2) in our GC cohort (Supplementary Fig. 5A), and their comparison with the COSMIC signatures found a high similarity with COSMIC Signature CN1, CN4, or CN9 (Supplementary Fig. 5B). Importantly, a significant difference was observed in CN9 (= Sig1) between Responders and Non-responders (Supplementary Fig. 5C, *P* = 0.029 by Mann–Whitney U test). CN9 (= Sig1) has been reported to be positively associated with an increase in leukocyte fraction, elevated hypoxia scores, *TP53* mutations, and *PTEN* mutations in specific cancers [34] [35] [36].

### Differential expressions of immune-related genes is associated with chemotherapy response

**Fig. 2:**
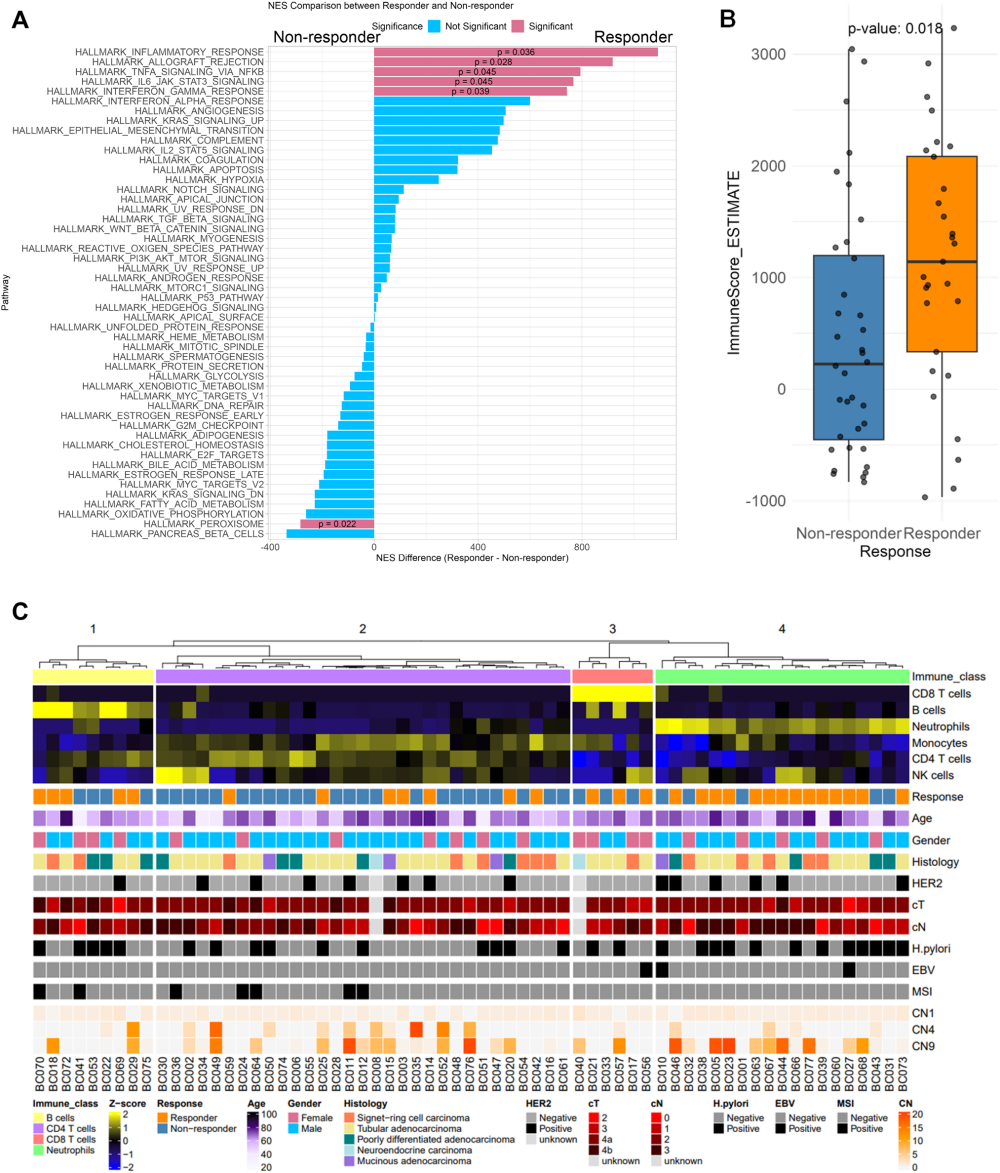
Multi-omics data from a cohort of 65 GC patients. **A** Comparison of the HALLMARK dataset between Responders and Non-responders. **B** Comparison of tumor microenvironment signature scores between Responders and Non-responders. **C** Immunological signatures, clinical data, and copy number signatures detected by targeted sequencing in 65 GC patients. These clusters are categorized based on the clustering of immune signatures

In RNA expression profiles of GCs from Responders and Non-responders, variability was observed in 492 genes; however, no specific pathways were identified using ReactomePA (Supplementary Figs. 6, and Supplementary Table 4). To investigate whether specific gene sets exhibited statistically significant expression changes between Responders and Non-responders, we conducted gene set enrichment analysis (GSEA). Utilizing the HALLMARK dataset and ImmuneScore (Fig 2A and B), we observed a significantly difference of the expression variations in the gene set below: HALLMARK_IL6_JAK_STAT3_SIGNALING (*P* = 0.045), HALLMARK_INFLAMMATORY_RESPONSE (*P* = 0.036) and HALLMARK_INTERFERON_GAMMA_RESPONSE (*P* = 0.039), ImmuneScoreESTIMATE (*P* = 0.018). Notably, many immune-related and inflammation-related signatures showed significant differences between Responder and Non-responders of GC.

We previously reported that immune signatures and CNS differed between chemotherapy Responders and Non-responders in esophageal squamous cell carcinoma (ESCC) [37]. Therefore, we applied these approaches to this GC cohort. The CIBERSORT [38] deconvolution method, using the LM6 reference [39], classified these GCs into four immune subgroups (B cells, CD4 + T cells, CD8 + T cells, Neutrophils) (Fig. 2C). Interestingly, the Neutrophil group (*P* = 0.002) had more Responders, while the CD4 + T cells group (*P* = 0.001) had more Non-responders (Fig. 2C and Supplementary Fig. 7A). Clinical information showed no difference between Responders and Non-responders, nor among the respective subclusters (Fig. 2C). No significant changes were observed in the signatures of Neutrophils and CD4 + T cells based on histological type (*P* > 0.05, Supplementary Fig. 7B). Additionally, no significant relationship was found between various chemotherapy regimens and neutrophil signatures (*P* > 0.05, Supplementary Fig. 7C). Furthermore, one CNS CN9 (= Sig1) was significantly higher in the Neutrophil subgroup of immune signatures (Fig. 2B, and Supplementary Fig. 7D, *P* = 0.047 by Mann–Whitney U test). Comparing the 6-month survival rate (Supplementary Fig. 8A) and PFS (Supplementary Fig. 8B) of each immune subgroup, the Neutrophil subgroup had a better survival period compared to the other groups (Supplementary Fig. 8A: *P* = 0.044 by log rank test). These results suggest that immune signatures, neutrophil signatures, and CNS could influence the responsiveness of chemotherapy in GC.

### Predicting the chemotherapy effectiveness by machine learning approach

**Fig. 3:**
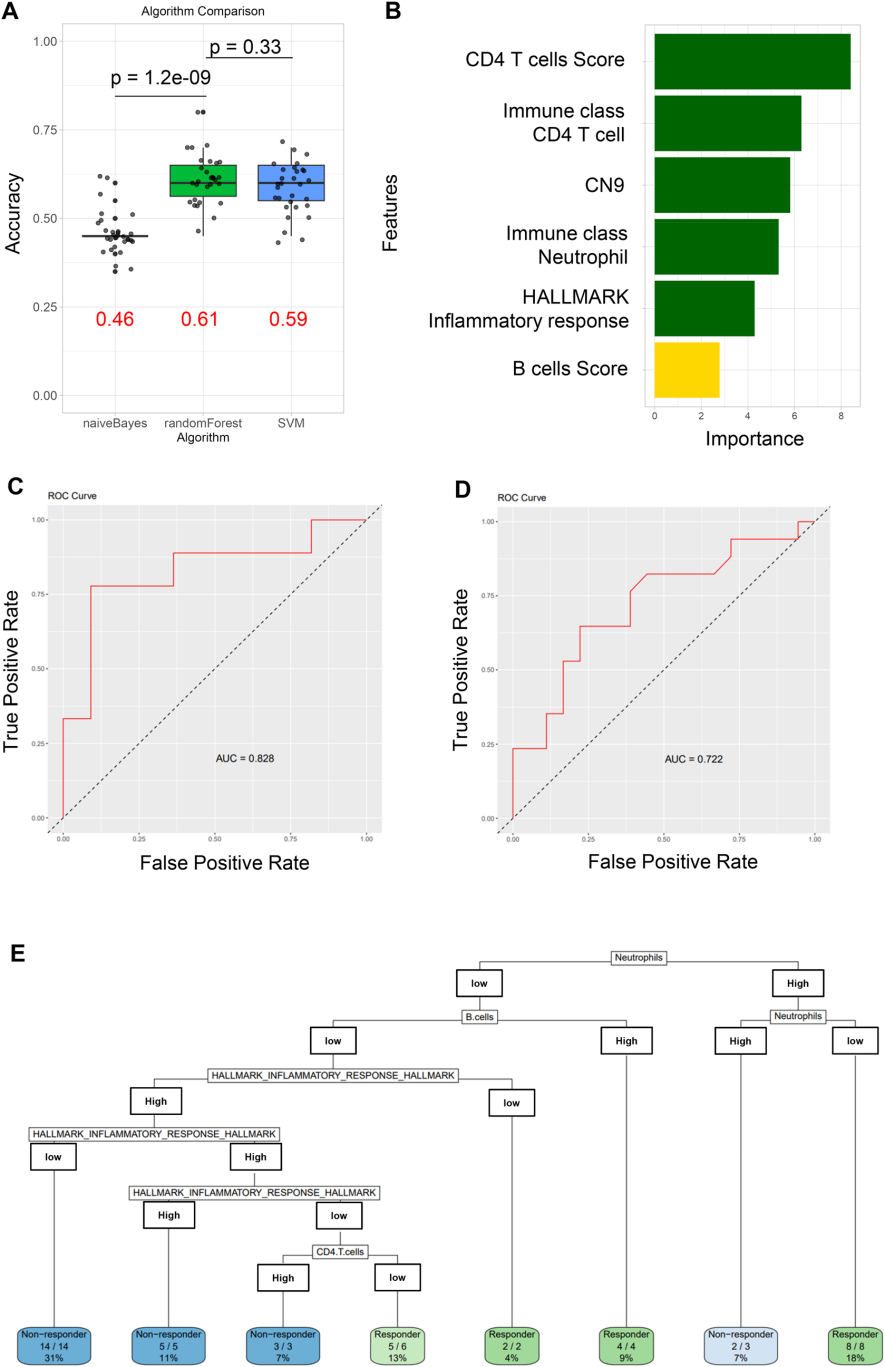
Prediction of chemotherapy responsiveness in GC patients using machine learning approach. **A** Selection of machine learning models. We performed evaluations using Random Forest, SVM, and Naïve Bayes, and compared their accuracies. **B** Using the Boruta algorithm, six key factors were identified as important out of the 123 features. **C ** The model was developed using 70% of the dataset and evaluated with the remaining 30% as the test data. AUC and ROC curve on the test data is shown. **D** AUC and ROC curve on the independent validation data from a Chinese GC cohort. **E** Random Forest, as an ensemble of multiple decision trees, was used to generate decision trees, and the tree most closely aligned with the Random Forest results was selected for further analysis

Sundar et al. demonstrated that a Random Forest method could classify GC patients into Responders and Non-responders based on a 19-gene signature [40]. Interestingly, among the genes they identified, five (*PTPRC, CD3G, TBX21, IL17A, FCGR3A*) are associated with CD4+ T cells and neutrophils. Therefore, we hypothesized that several factors, including neutrophils, could have a complex impact on the efficacy of chemotherapy, and that a machine learning approach could be utilized to predict the effects of chemotherapy. To select a machine learning method, we initially performed 30 simulations by dividing integrated data with 123 of factors into 70% training and 30% test data using three methods: Random Forest, SVM (support vector machine), and Naïve Bayes, to measure accuracy. The results showed accuracies of Random Forest: 0.61, SVM: 0.59, Naïve Bayes: 0.46, leading us to choose Random Forest (Fig. 3A). To improve the predictive accuracy of the machine learning model, we aimed to select important features and eliminate irrelevant or redundant ones using the R package Boruta [41], evaluating the importance of all features (123 factors of genomics, RNA expression, signatures, and clinical information) through 10,000 simulations (Fig. 3B, Supplementary Fig. 8, Supplementary Table 5). As a result, immune clusters of neutrophils and CD4 + T cells, immune cell signatures of CD4 + T cells and B cells, INFLAMMATORY RESPONSE, and one CNS, CN9, were identified as important factors related to chemotherapy response in GC. Since the immune clusters reflect CIBERSORT scores, it was also suggested that the immune signature of neutrophils could be a key factor associated with chemotherapy response. Using these key factors as continuous variables, we constructed a diagnostic model to predict chemotherapy response using the Random Forest method. The predictive diagnostic model showed an accuracy of 0.80 and an AUC of 0.82 on the test data, with an AUC of 0.85 on the training set (Fig. 3C). Furthermore, we also ran this algorism on the data set of another cohort of GC patients (Ziyu Li et al. 2020 [33]. n = 35), which performed genomic and RNAseq of GC samples in China and analyzed the correlation of combination chemotherapy, and tested our predictive diagnostic model as validation dataset. The results for the Chinese cohort showed an AUC of 0.72 (Fig. 3D). Figure 3E illustrates the visualization of this diagnostic predictive model.

According to this model in Fig. 3E, neutrophils are the most critical factor to predict the response; a certain level of neutrophil presence in GC tissues increases chemotherapy sensitivity, while excessive infiltration leads to poor chemotherapy response (Fig. 3E, right branch). Also, the presence of B cells with minimal neutrophil infiltration predicted higher chemotherapy sensitivity. Conversely, a low presence of neutrophils with a high inflammatory response in cancer cells predicted poorer response. However, under these conditions, a lower presence of CD4 + T cells predicted better responsiveness. These results suggest that one reason for poor responsiveness in GC may be increased inflammatory response in the tumor microenvironment (TME) due to the presence of CD4+ T cells. Interestingly, the presence and balance of neutrophils were suggested to potentially influence chemotherapy responsiveness in GC.

### Molecular characterization of neutrophil subclusters by single-cell RNAseq

As the next step, we focused on neutrophils in the tumor microenvironment (TME) of GC, which is one of the critical factors for predicting chemotherapy response in this study. Tumor-associated neutrophils (TANs) are a type of white blood cell present in the TME and play complex and diverse roles in cancer progression, metastasis, and immune response. The function of TANs in promoting or inhibiting tumor growth and metastasis depends on the TME. In our previous study, we reported that neutrophils infiltrating ESCC had a pro-tumor function that negatively impacted chemotherapy responsiveness [37]. In contrast, in this study, neutrophils infiltrating GC appeared to have an inhibitory function that positively affected chemotherapy responsiveness. By comparing this dual role, we hypothesized that characterizing neutrophils and other immune cells in TME could provide insights into their roles. We extracted CD45 + immune cells infiltrating tumor tissues from four ESCC patients [37] and four GC patients and performed sc-RNAseq. After filtering out low-quality cells and doublets (details in Methods), we annotated 59,584 immune cells infiltrating GC and ESCC using SingleR (Supplementary Fig. 9A).

**Fig. 4:**
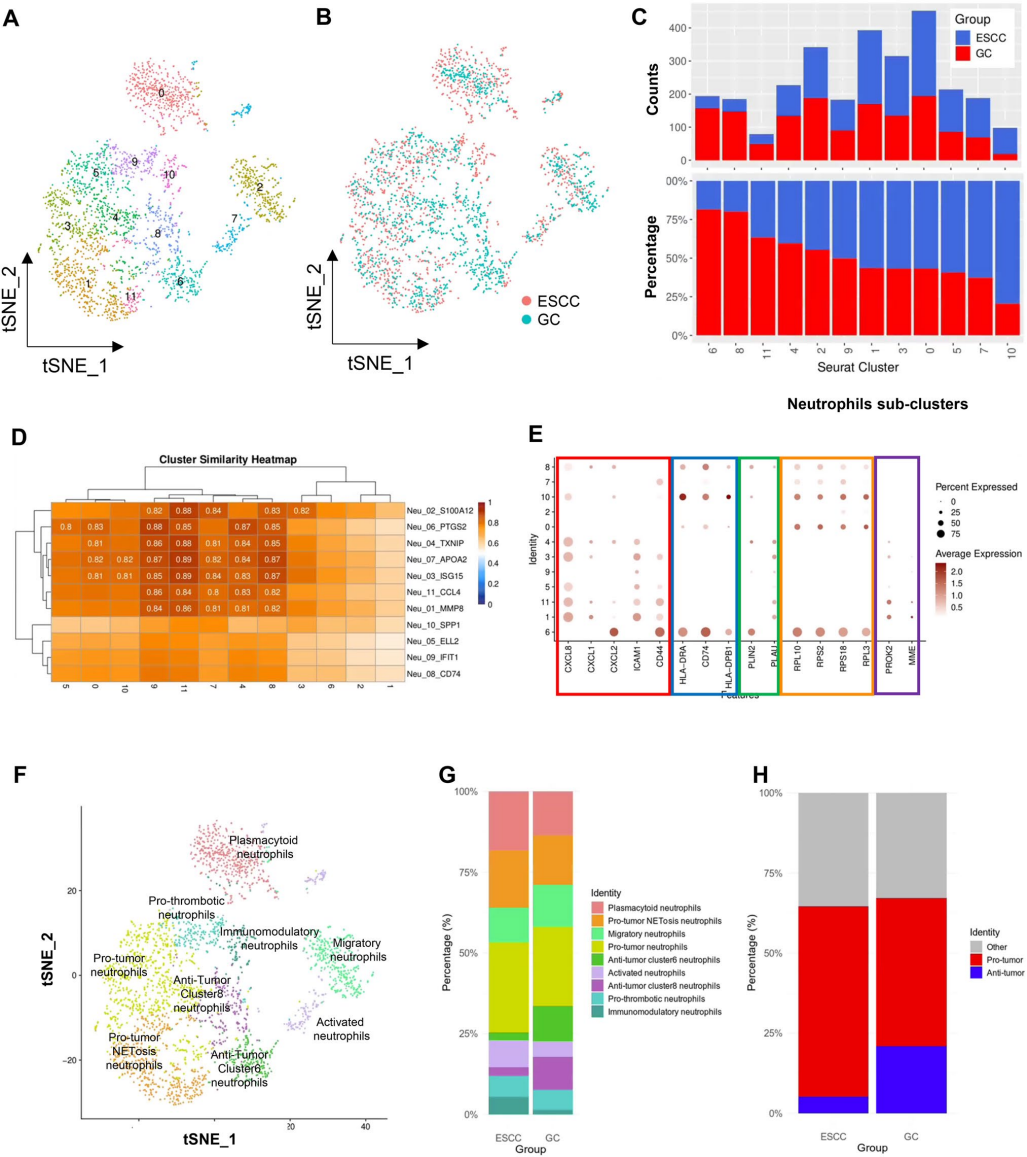
Single-cell RNA-seq analysis of neutrophils retrieved from GC and ESCC tissues. **A** t-SNE shows 12 sub-clusters of neutrophils retrieved from GC tissues (this study) and ESCC tissues (our previous study [37]). **B** t-SNE of neutrophils from ESCC and GC tissues, color-coded accordingly. **C** The number and percentage of immune cells from GC and ESCC tissues in each subclass. **D** The similarity between previously reported subclusters of neutrophils infiltrating the liver and those infiltrating gastrointestinal cancers. **E** Comparison of expression levels of gene markers in the clusters: TAN1 (*CXCL8, CXCL1, CXCL2, ICAM1, CD44*), TAN2 (*HLA-DRA, CD74, HLA-DPB1*), TAN3 (*PLIN2, PLAU*), TAN4 (*RPL10, PRS2, RPS18, RPL3*), and NETosis (*PROK2, MME*). **F** Annotation of neutrophils. **G** Stacked bar graph showing the distribution of annotated neutrophils in GC and ESCC. **H** Stacked bar graph categorizing neutrophils into Pro-tumor, Anti-tumor, and Other

Focusing on neutrophils, which are difficult to select and annotate using conventional methods [42], we used two approaches: a modified method by 10x Genomics and the method by Roudong Xue et al. [23]. As a result, we identified 12 subclusters of 3,822 neutrophils (Supplementary Fig. 9B), but Cluster 10 included clusters with a high frequency of immunoglobulins such as *IGV3-1* (Supplementary Fig. 9C). Therefore, we excluded clusters or cells with a high frequency of immunoglobulins or those expressing suspicious lineage marker genes such as *CD3E*, *CD19*, *NCAM1*, *CD8A*, and *CD4* (Supplementary Fig. 9E). We finalized these neutrophils formed from 12 subclusters, as shown in Fig. 4A**. **Figure 4B shows the distribution by GC or ESCC tissues and Supplementary Fig. 9E shows the distribution of anonymized samples. Analysis of the cancer type distribution in these subclusters revealed that Clusters 6 and 8 had a higher proportion of neutrophils infiltrating GC, while Cluster 10 had more neutrophils infiltrating ESCC (Fig. 4C).

Next, to characterize the 12 subclusters, we evaluated whether our cohort's neutrophil subclusters aligned with those reported in previous studies that documented the heterogeneity of neutrophils [42]. The heatmap in Fig. 4D shows the similarity between our cohort's neutrophil subclusters and those determined by the previous study [23]. Most clusters had a similarity of over 60%, suggesting that the neutrophils identified by SingleR and filtering were correctly selected. Interestingly, although many of the neutrophils infiltrating GC and ESCC showed similarities with those determined by the previous study [23], they were not distinctly separable as clusters and potentially exhibited complex combined characteristics (Fig. 4D). Moreover, Clusters 1, 2, and 6 were suggested to have unique features specific to GC or ESCC. Therefore, we considered it necessary to create a unique sub-classification of neutrophils for GC. Since classification markers for some TANs have been reported [24] [25] [16], we investigated genes related to these markers (Fig. 4E) and performed enrichment analysis using ReactomePA on genes specifically expressed in each cluster to identify pathways represented by each signature (Supplementary Fig. 10).

Clusters expressing TAN1 gene markers (*CXCL1, CXCL2, CXCL8, ICAM1, CD44)*, associated with neutrophil activation, recruitment, cell adhesion, and NET formation, were found in Clusters 1, 3, 4, 5, and 11. Clusters strongly expressing TAN2 gene markers (*HLA-DRA, CD74, HLA-DPB1*, and other MHC II-related genes) associated with immunogenic antigen presentation and potential anti-tumor immunity were seen in Clusters 0, 6, 8, and 10. Clusters expressing TAN3 gene markers related to lipid metabolism (*PLIN2* and *PLPP3*), which potentially play roles in activating extracellular matrix proteases and mediating tumor cell adhesion and movement through interaction with the homologous receptor uPAR expressed on tumor cells, were observed in Clusters 6, 1, 11, 5, 9, 3, 4, 10, and 8. Clusters expressing TAN4 gene markers (ribosomal-related genes *RPL10, RPS2, RPS18, RPL3*), suggesting a potential transition to tumor-associated neutrophils, were found in Clusters 6, 0, 2, 10, 7, and 8. NETosis gene markers *PROK2* and *MME* were expressed in Clusters 1 and 11.

Based on these results, Cluster 0 showed high expression of immunoglobulin-related genes *IGHA1* and *IGKC*, commonly expressed in B cells and plasma cells (Supplementary Table 6), and was involved in pathways related to protein synthesis and translation activation (Supplementary Fig. 10), suggesting characteristics of plasmacytoid neutrophils. Clusters 1, 3, 4, 5, and 11 (especially 1 and 11, involved in NETosis) displayed pro-tumor TAN characteristics. Clusters 2 and 7 contained pathways related to mitochondria and RHO signaling (Supplementary Fig. 10), suggesting that Clusters 2 and 7 may be activated neutrophils involved in promoting cell migration and motility (other pathways defined Cluster 2 as Migratory and Cluster 7 as Activated). Clusters 6 and 8 showed TAN2 markers and interleukin-related pathways, exhibiting anti-tumor TAN characteristics. Interestingly, Clusters 6 and 8 were prevalent in GC patients and, due to their clustering distance in Seurat dot plots (Fig. 4E), were suggested to be distinct types of neutrophils with different characteristics.

Cluster 9 had multiple pathways related to platelet activation, signaling and aggregation, thrombin signaling through proteinase-activated receptors (PARs), and thromboxane signaling through the TP receptor (Supplementary Fig. 10), suggesting pro-thrombotic neutrophil characteristics. Cluster 10, which included pathways involved in immune response regulation, cytokine signaling, and antiviral responses, had features of immunoregulatory neutrophils. These characteristics are summarized in Fig. 4F. The proportion of these annotated neutrophils in GC and ESCC (Fig. 4G) was analyzed, and the characteristics were grouped into pro-tumor, anti-tumor, and other categories, showing their proportions (Fig. 4H). The results indicated that GC contained a higher proportion of anti-tumor neutrophils, suggesting that the differences in neutrophil ratios could contribute to the chemotherapy responsiveness of GC and ESCC.

### Neutrophil signature is related to chemotherapy response and survival of GC

Next, we investigated whether these specific types of neutrophils are actually present in stomach tissue by using spatial transcriptome (ST) data of human gastric mucosa: stomach encyclopedia [26], and cell annotations were performed using the method described by Tsubosaka et al. [26]. From two ST datasets, we extracted 2,316 neutrophil fractions and created signatures for each of the neutrophil clusters identified in Fig. 4E using cluster-specific genes (Supplementary Table 7, and Supplementary Fig. 11). We then confirmed that these anti-tumor neutrophils were actually present within stomach tissues (Supplementary Fig. 12A). Additionally, we extracted neutrophil fractions from the ST data, identifying 463 pro-tumor neutrophils, 179 anti-tumor cluster 6 neutrophils, and other fractions, and displayed them using t-SNE (Supplementary Fig. 12B). However, Anti-Tumor Cluster 8 Neutrophils could not be confirmed due to the absence of detectable cluster-specific genes in the ST data.

**Fig. 5:**
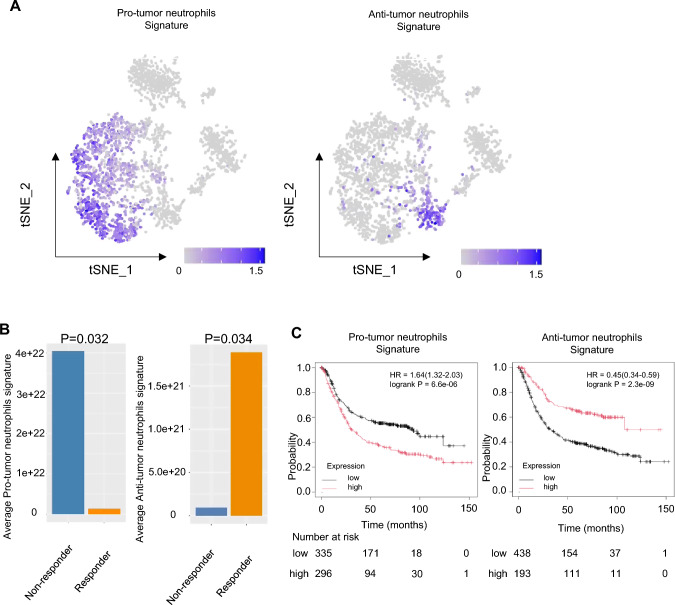
Pro-tumorigenic and anti-tumorigenic neutrophil signatures. **A** Feature plots depicting cells expressing Pro-tumor neutrophil (left) and Anti-tumor cluster 6 neutrophil (right) signatures in single-cell RNAseq data. **B** Comparison of Pro-tumor neutrophil (left) and Anti-tumor cluster 6 neutrophil (right) signatures between Responders and Non-responders in bulk RNA-seq data. **C** Survival curves in a large cohort (*N* = 631) for Pro-tumor neutrophil (left) and Anti-tumor cluster 6 neutrophil (right) signatures

After confirming the presence of Pro-tumor Neutrophils and Anti-Tumor Cluster 6 Neutrophils within stomach tissues, we created signatures using genes specifically expressed in these neutrophil groups (Fig. 5A**,** and Supplementary Table 8). Comparing these signatures within our GC cohort, we found that pro-tumor neutrophils showed significantly higher signals in Non-responders compared to Responders, while anti-tumor cluster 6 neutrophils showed significantly higher signals in Responders (Fig. 5B; pro-tumor neutrophils: *P* = 0.032, anti-tumor cluster 6 neutrophils: *P* = 0.034, Mann–Whitney test). Although data were limited and significant differences in survival and PFS could not be detected (Supplementary Fig. 13), these signatures were significantly associated with clinical outcomes in another clinical GC study [43] where only samples with available data for selected probes measured by the HGU133 plus 2.0 array were analyzed (Fig. 5C, *N*= 631; pro-tumor neutrophils signature: *P* = 6.6e-06, anti-tumor neutrophils signature: *P* = 2.3e-09). These results suggest that neutrophils with Pro-tumor or Anti-tumor characteristics may provide a synergistic benefit to patients, both alone and in combination with chemotherapy. Notably, in GC, the presence of a higher proportion of Anti-tumor neutrophils led to a greater number of patients in the immune cluster being classified as Responders. Conversely, in our previous study on ESCC, a higher proportion of Pro-tumor neutrophils resulted in more patients in the immune cluster being classified as Non-responders.

## Discussion

In our investigation, we successfully developed a diagnostic model capable of predicting the response of GC patients to chemotherapy. By integrating a comprehensive set of 123 factors, including clinical data, genomic profiles, immune signatures, and copy number variations, this model represents a significant advancement in personalized medicine. Utilizing data from a cohort of Japanese patients, with 70% for training and the remaining 30% for testing, our model achieved an impressive 80% accuracy rate in predicting chemotherapy outcomes. This performance was further validated in a Chinese GC patient dataset [33], where the model maintained over 70% accuracy. The incorporation of immune signatures, clinical information, and genomic data underscores the potential of machine learning in refining therapeutic strategies not only for GC but also for ESCC.

A key finding of this study is the identification of neutrophils as a major determinant of chemotherapy responsiveness. Our research revealed the complex roles of neutrophils in GC, which diverges from previous understandings derived mainly from ESCC studies. Through the analysis of TAN markers, we identified 11 distinct neutrophil subclusters within GC tissues, which exhibited diverse roles ranging from promoting tumor growth to exhibiting anti-tumor functions. Notably, Clusters 1, 3, 4, 5, and 11 were associated with pro-tumor activities, with Clusters 1 and 11 particularly involved in the formation of NETs, which contribute to cancer progression and metastasis. In contrast, Cluster 6 was found to demonstrate anti-tumor functions. The presence of these neutrophil types was confirmed in ST data from another cohort.

Our study also underscores the prognostic value of neutrophil signatures in GC patients. The analysis of a large cohort indicated that patients with high anti-tumor neutrophil signatures had longer survival times, while those with high pro-tumor neutrophil signatures had shorter survival times. In our cohort, these neutrophil signatures were shown to serve as indicators of chemotherapy responsiveness. Additionally, our previous study on ESCC demonstrated that the removal of neutrophils improved chemotherapy responsiveness, suggesting that the removal of pro-tumor neutrophils identified in this study may have contributed to enhanced chemotherapy outcomes. This highlights the potential of neutrophil profiles as predictive biomarkers for the effectiveness of chemotherapy.

However, it is important to acknowledge the limitations of our study. Although the predictive model is robust, it is primarily based on data from Asian populations, which may limit its applicability to other ethnic groups without further validation. Additionally, the validation cohort received neoadjuvant chemotherapy (NAC), whereas the patients in this study underwent palliative chemotherapy. These represent different patient populations, and this should be considered a limitation of our study. Moreover, the neutrophils detected in the ST data were not identified using all available genes. Furthermore, the complexity of the tumor microenvironment and the heterogeneity of GC necessitate more detailed studies to fully elucidate the mechanisms by which neutrophils influence chemotherapy responsiveness.

To translate our findings into clinical practice, extensive validation in diverse patient populations will be necessary. Future research should focus on exploring the therapeutic potential of targeting specific neutrophil subclusters and understanding the mechanisms by which neutrophils impact chemotherapy outcomes. Ultimately, our work paves the way for more personalized and effective treatment strategies for GC, emphasizing the crucial role of the immune system in cancer therapy.

## Conclusions

This study demonstrates the utility of machine learning in advancing personalized medicine for gastric cancer. It identifies tumor-associated neutrophils and their subgroups as key predictors of chemotherapy response. These findings have the potential to lead to more tailored and effective treatment plans for patients.

## Supplementary Information

Below is the link to the electronic supplementary material.

Supplementary file1 (XLSX 12 KB)

Supplementary file2 (PDF 2427 KB)

## Data Availability

All authors agree to share the data related to current study. WGS and RNA-seq reads for matched tumor, and non-tumor tissues data have been deposited at the National Bioscience Database Center (NBDC) and are publicly available under accession number JGAD000895. The sc-RNAseq data has been deposited to DDBJ BioProject database under accession number PRJDB15365.

## Abbreviations

AUC: Area under the curve
CIN: Chromosomal instability
cN: Clinical nodal stage
CNAs: Copy number alterations
CNS: Copy number signatures
CNVs: Copy number variations
COSMIC: Catalogue of Somatic Mutations In Cancer
CR: Complete response
cT: Clinical tumor stage
EBV: Epstein–Barr virus
ESCC: Esophageal squamous cell carcinoma
FP: Fluoropyrimidines + Platinum
FPKM-UQ: Fragments Per Kilobase of Exon per Million Fragments Mapped-Upper Quartile
GC: Gastric cancer
GISTIC: Genomic identification of significant targets in cancer
GSEA: Gene set enrichment analysis
GS: Genomically stable
HER2: Human epidermal growth factor receptor 2
MHC: Major histocompatibility complex
MSI: Microsatellite instability
NAC: Neoadjuvant chemotherapy
NETosis: A form of cell death that involves the release of neutrophil extracellular Traps (NETs)
NETs: Neutrophil extracellular traps
PD: Progressive disease
PFS: Progression-free survival
PGx: Pharmacogenomics
PR: Partial response
PTX: Paclitaxel
RPKM: Reads per kilobase of transcript per million mapped reads
RNAseq: RNA sequencing
SVM: Support vector machine
SD: Stable disease
ST: Spatial transcriptome
TANs: Tumor-associated neutrophils
TME: Tumor microenvironment
