# Supplementary material for: Predicting chemotherapy responsiveness in gastric cancer through machine learning analysis of genome, immune, and neutrophil signatures
Source: Gastric Cancer. 2024 Dec 2;28(2):228–44. doi: 10.1007/s10120-024-01569-4 (PMC11842519; doi:10.1007/s10120-024-01569-4)
Supplement: Supplementary file 2 — Supplementary file2 (PDF 2427 KB) [file 10120_2024_1569_MOESM2_ESM.pdf]

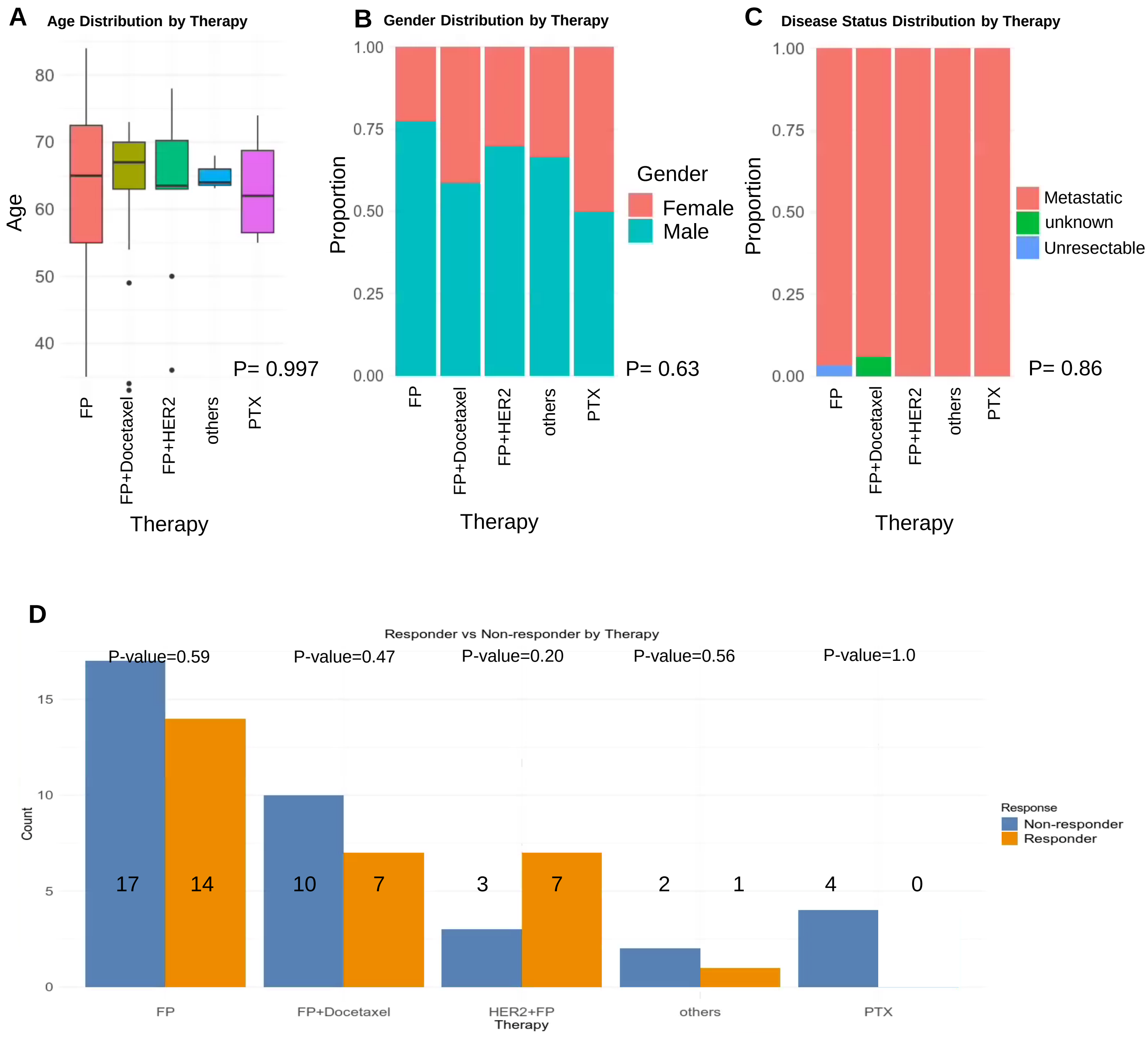

Supplementary Figure 1: Comparison of chemotherapy regimens and response rates in this GC cohort.

Patients who received chemotherapy were categorized into five groups: FP, FP+docetaxel, PTX, FP+HER2 (Trastuzumab), and others. (A) Comparison of patient age across the different chemotherapy regimens showed no significant differences (p-value= 0.99 by ANOVA test). (B) Gender distribution comparison among the groups also showed no significant differences (p-value = 0.63 by Pearson’s Chi-squared test). © Comparison of disease status among the groups indicated no significant differences (p-value = 0.86 by Pearson’s Chi-squared test). (D) Differences in response rates among the treatment regimens were not observed (P-values: FP = 0.56, FP+docetaxel = 0.47, PTX = 1.0, FP+ HER2 = 0.20, and others = 0.56 by Chi-squared test).

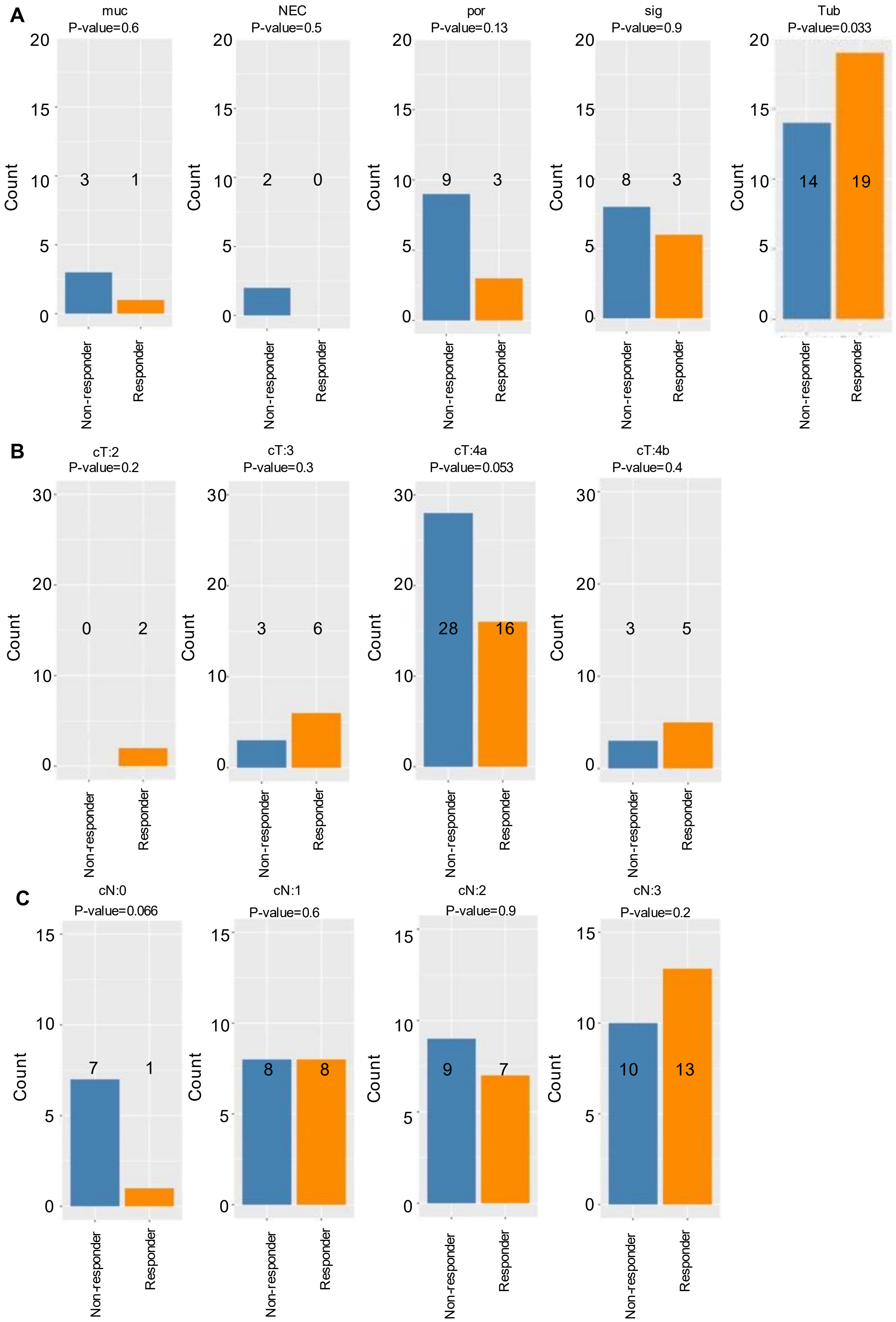

Supplementary Figure 2

Supplementary Figure 2: The histological classification and assessment of spread and progression in GC.

Each graph compares the histological classification (A), the extent (B) and progression(C) of GC between chemotherapy Responders and Non-responders. P-values were calculated using Fisher's exact test, and the numbers in the center of the graphs represent the number of patients. Abbreviations are as follows: Muc: Mucinous adenocarcinoma, Por: Poorly differentiated adenocarcinoma, Sig: Signet ring cell carcinoma, Tub: Tubular adenocarcinoma, NEC: Neuroendocrine carcinoma.

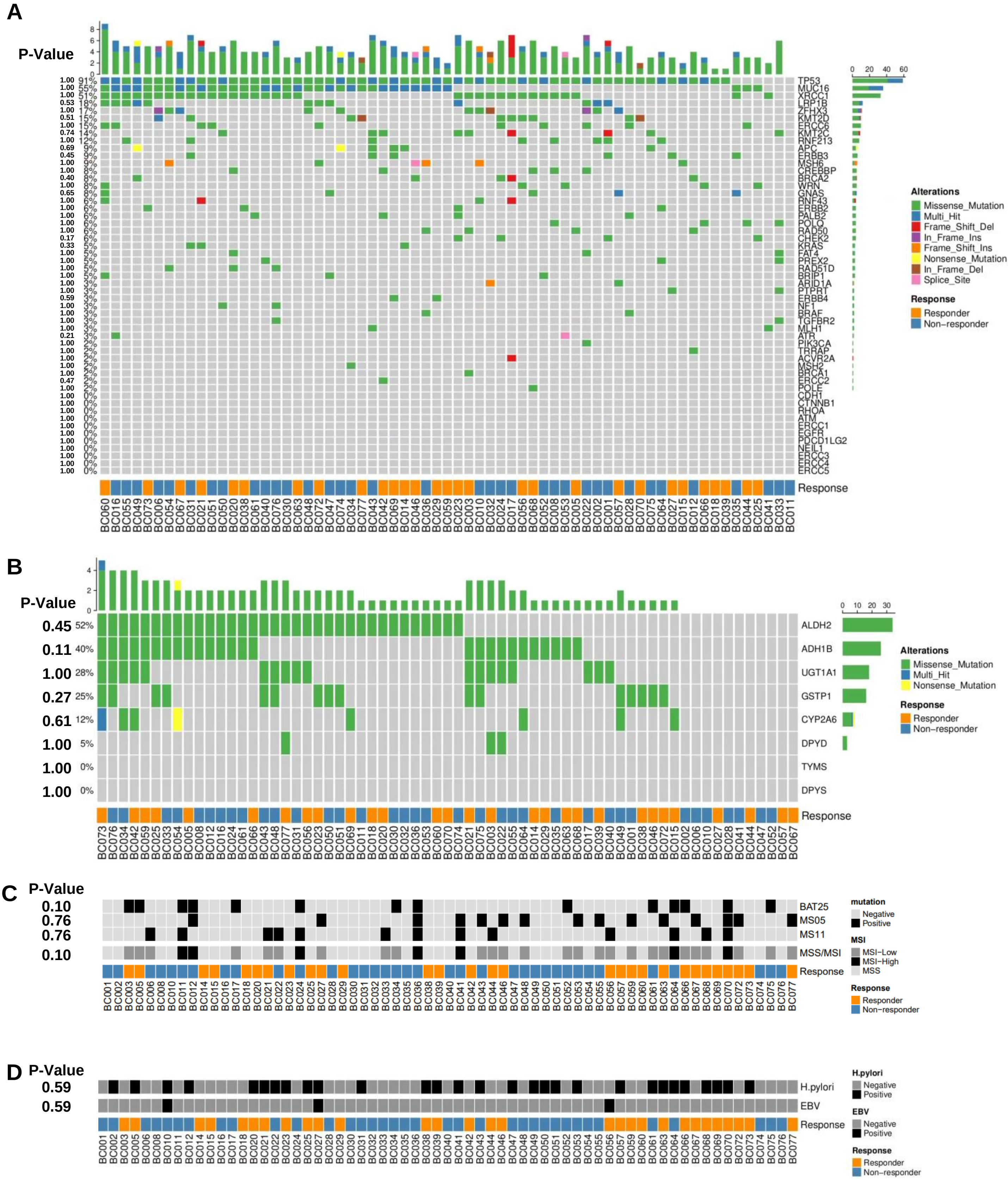

Supplementary Figure 3: Analysis of SNVs in GC patients using targeted deep sequencing. Display samples with somatic mutations: (A), germline mutations: (B), MSI: (C), and with Helicobacter pylori or EBV: (D) in Oncoplots.

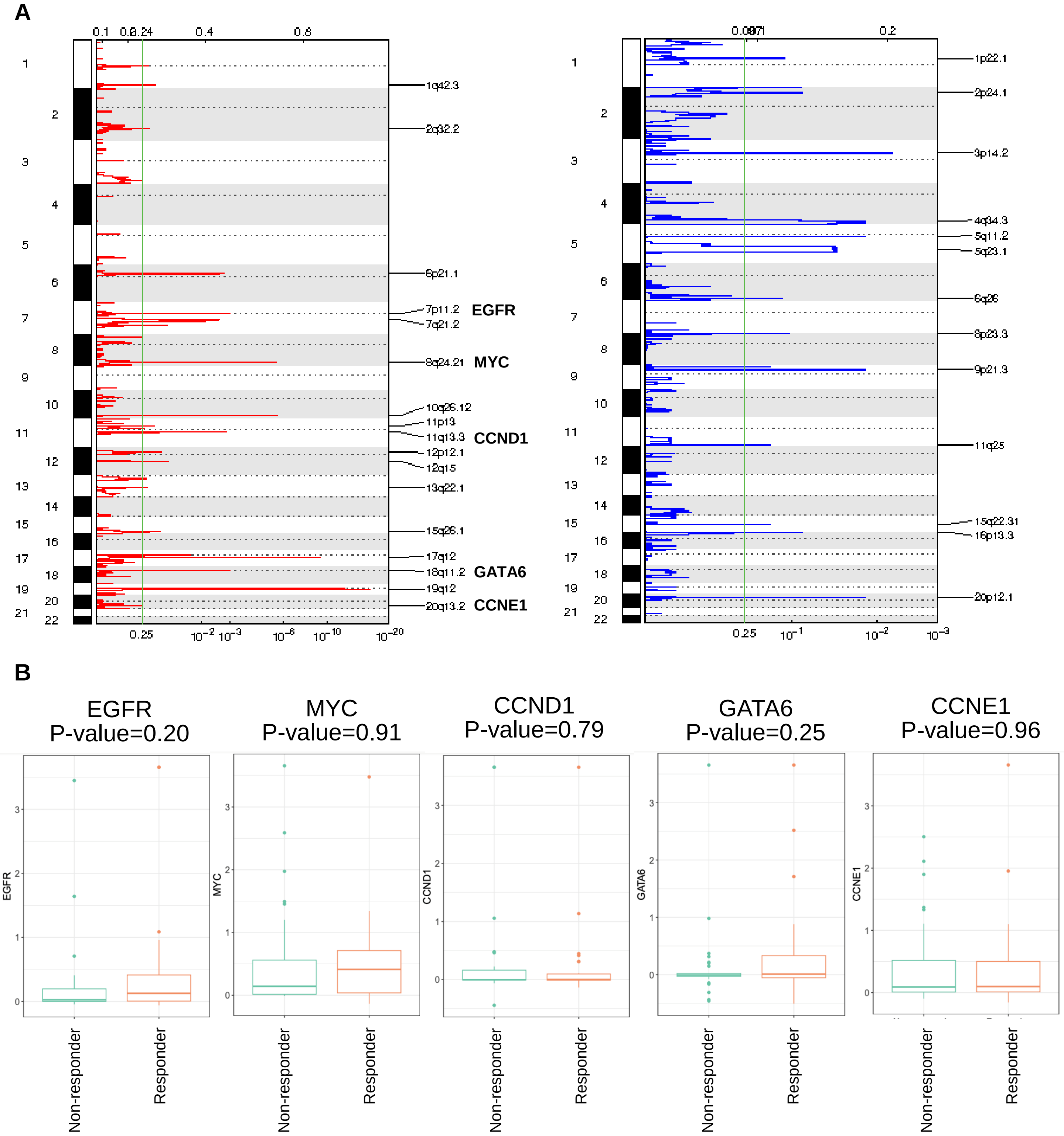

Supplementary Figure 4: Copy number analysis by GISTIC.
(A) Results of copy number analysis by GISTIC analysis. Red indicates gain, blue indicates loss. (B) Compared copy number variations in genes reported to be associated with GC between chemotherapy Responders and Non-responders.

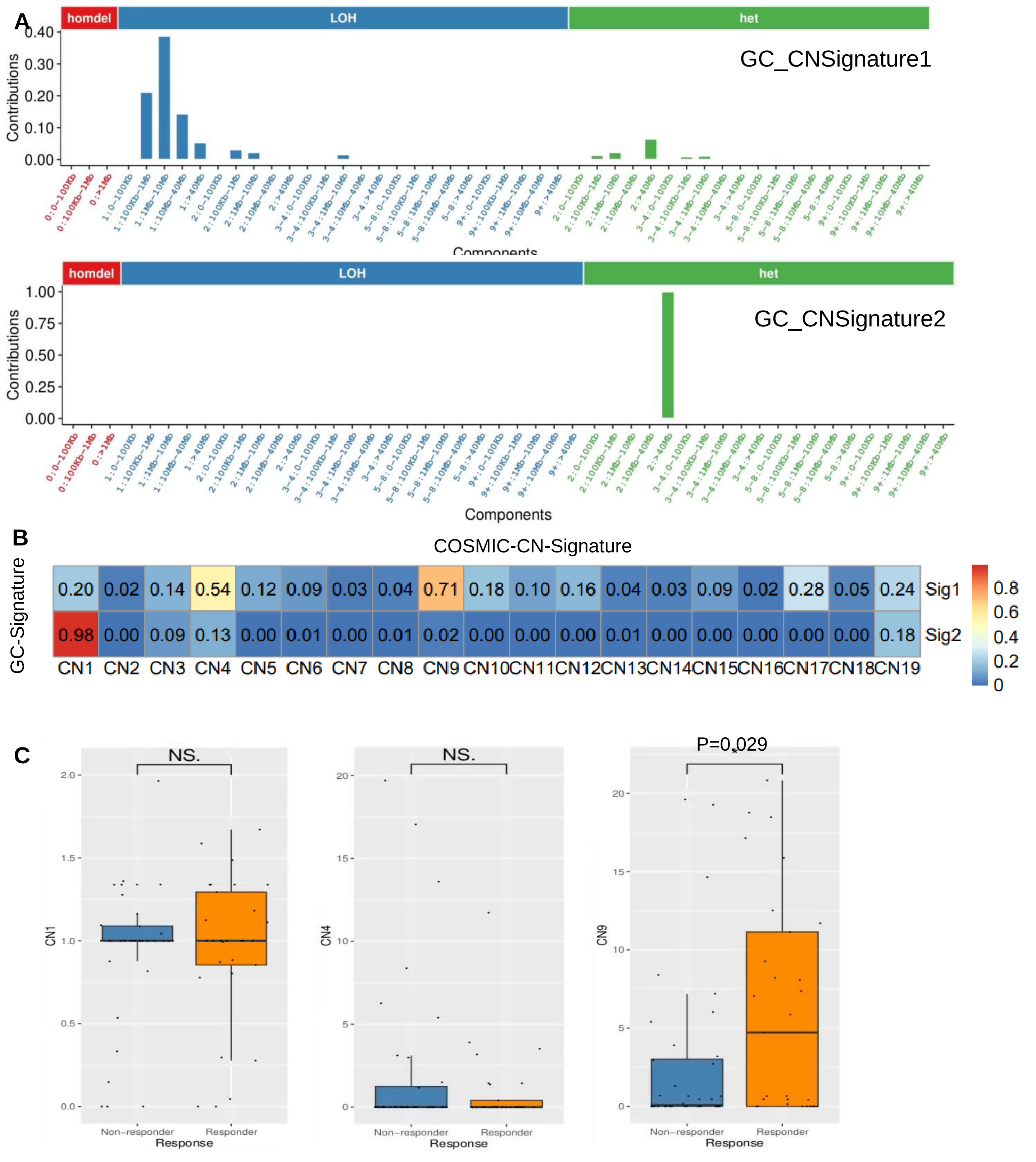

Supplementary Figure 5: Copy Number Signatures (CNS) in 65 GCs. (A) Upon calculating the copy number signatures (COSMIC) in GC patients, two types of signature patterns were detected. (B) Similarity between the two signature patterns and COSMIC signature patterns. (C) Comparison of COSMIC copy number signatures in chemotherapy Responders and Non-responders among. P-values were calculated using the Mann-Whitney test.

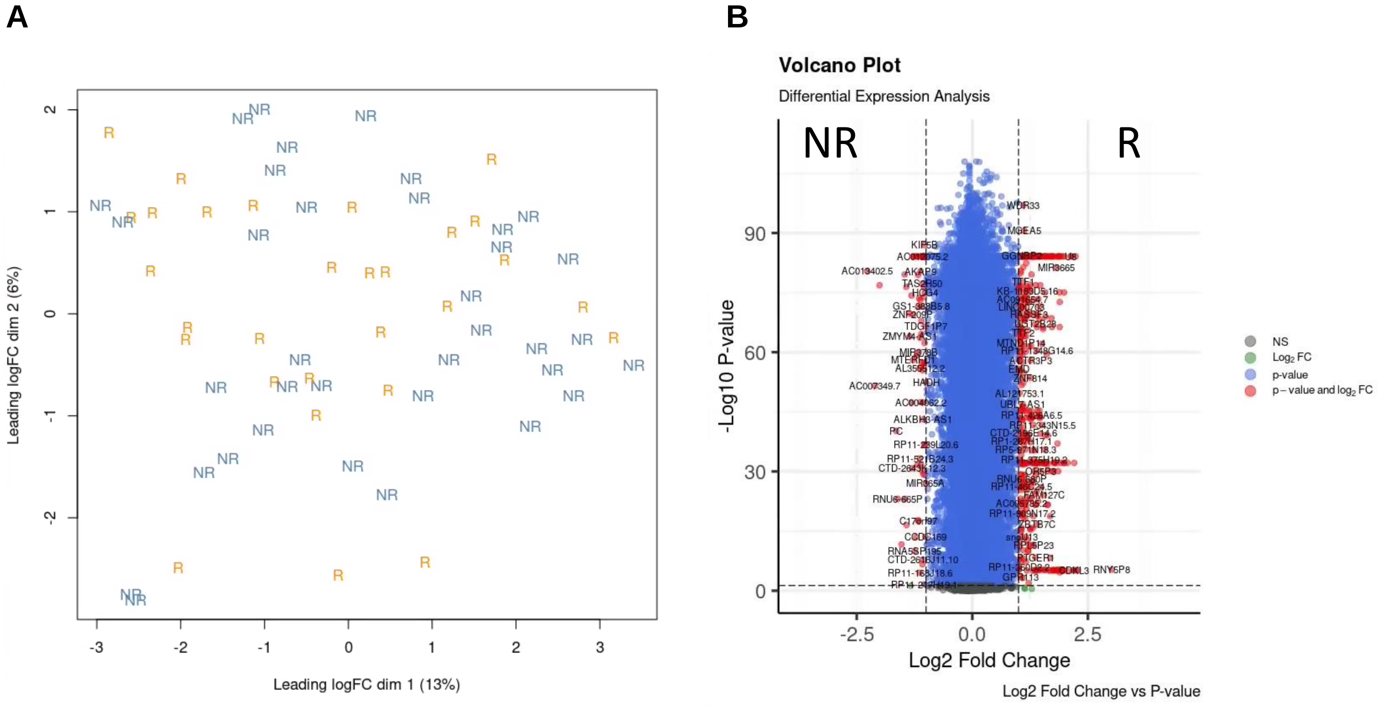

Supplementary Figure 6: Comparison of RNA expression changes by RNAseq of GC tissues.

(A) MDS plots show individual patients who are chemotherapy Responders (R) and Non-responders (NR). Responders and non-responders did not form distinct groups. (B) Volcano plot of differentially expressed genes in Responders and Non-responders.

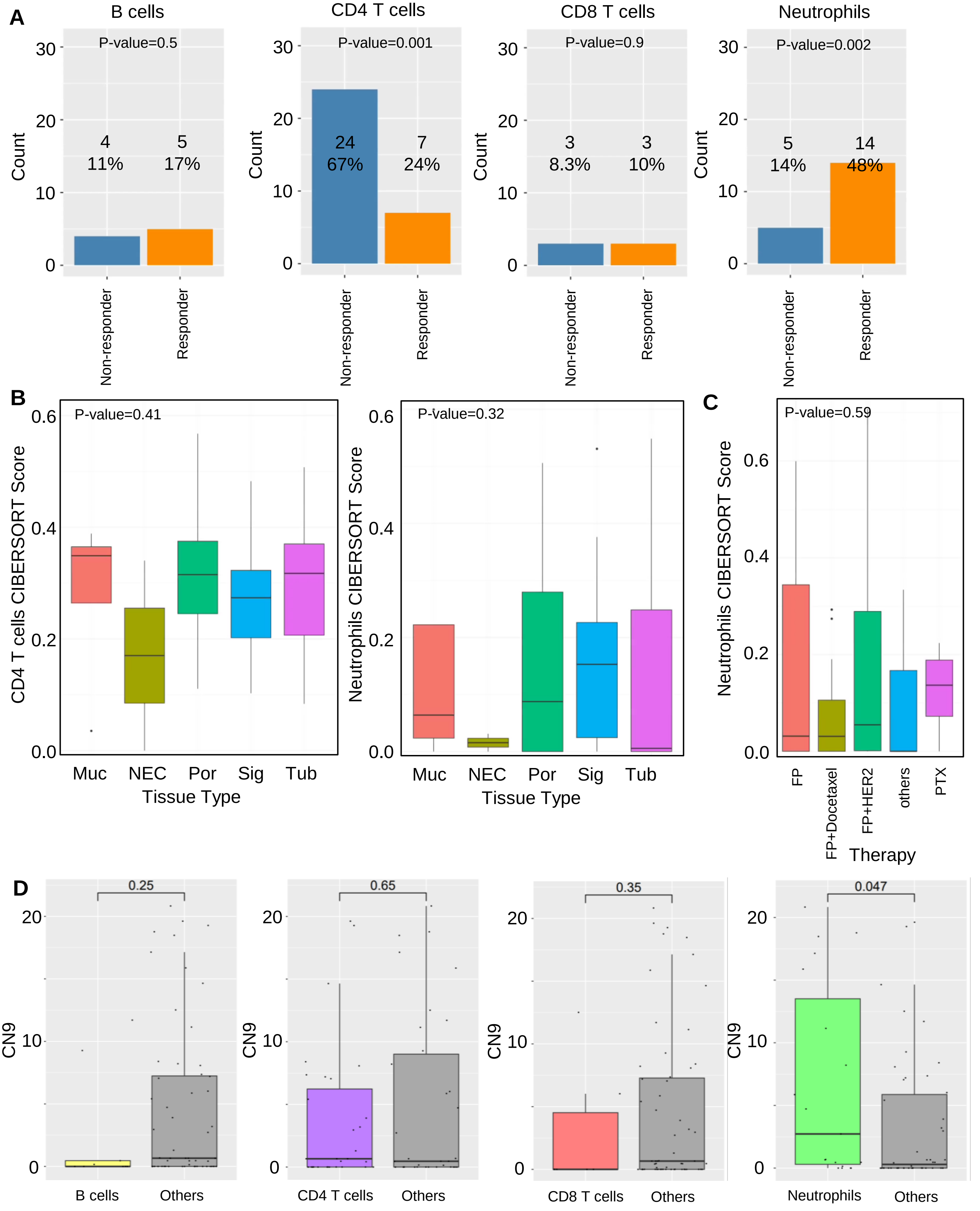

Supplementary Figure 7

Supplementary Figure 7: Comparison of chemotherapy Responders and Non-responders in the immune subclusters based on their immune signatures.

(A) The number of chemotherapy Responders and Non-responders in each cluster classified as B cells, CD4+ T cells, CD8+ T cells, and neutrophils. P-values were calculated using Fisher's exact test. (B) Comparison of CIBERSORT scores for CD4+ T cells (left) and neutrophils (right) across different histological types. (C) Comparison of CIBERSORT scores for neutrophils across different treatment regimens. (D) Comparison of copy number signatures between patients classified into the neutrophil group and other groups based on immune signatures. P-values were calculated using the Mann-Whitney test.

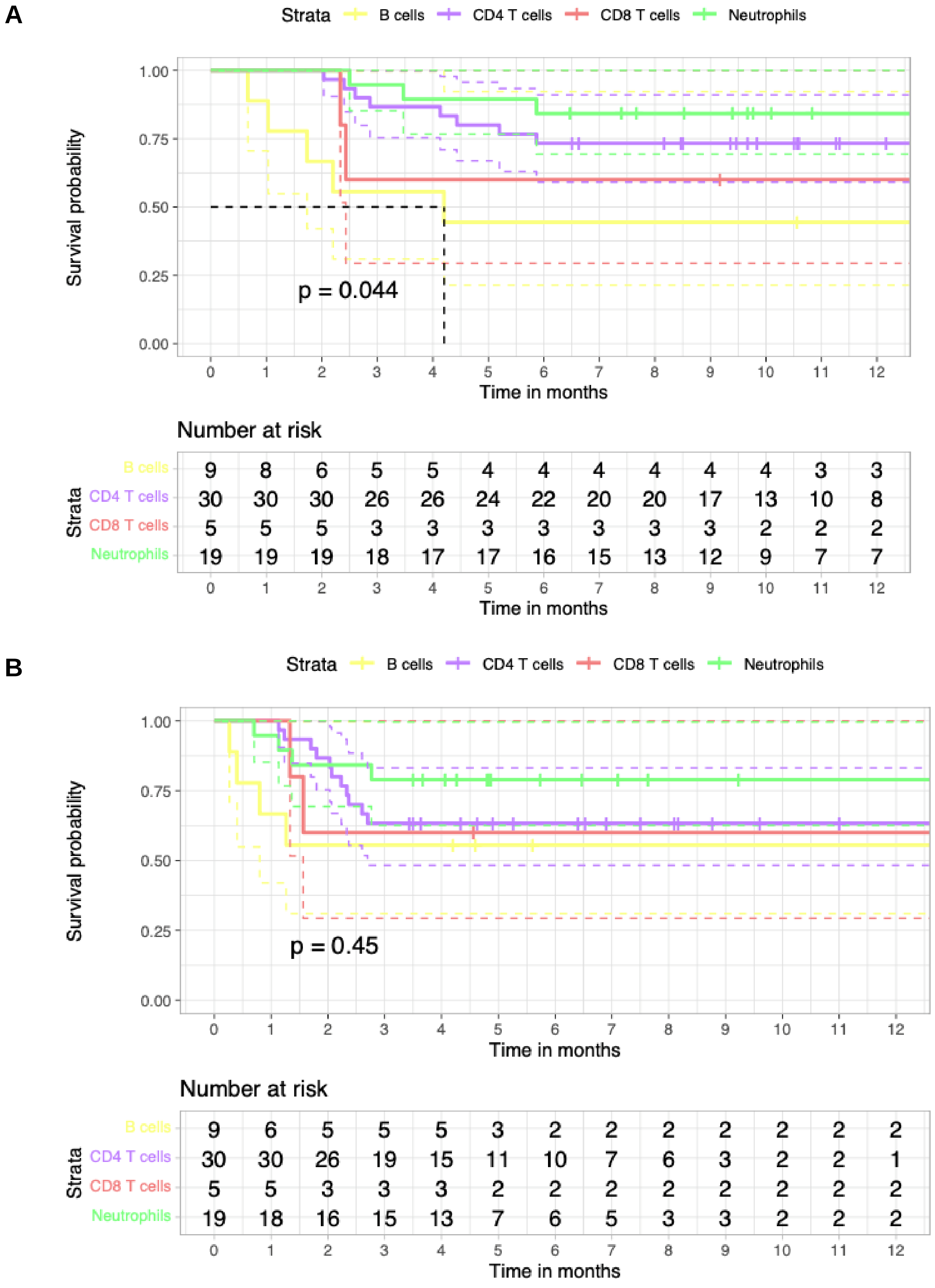

Supplementary Figure 8: Kaplan-Meier plots for classification of the immune subgroups. (A)Survival curves and (B) Progression-Free Survival (PFS) in four subgroups (B cells, CD4+ T cells, CD8+ T cells, Neutrophils) classified based on the immune signatures of GC tissues.

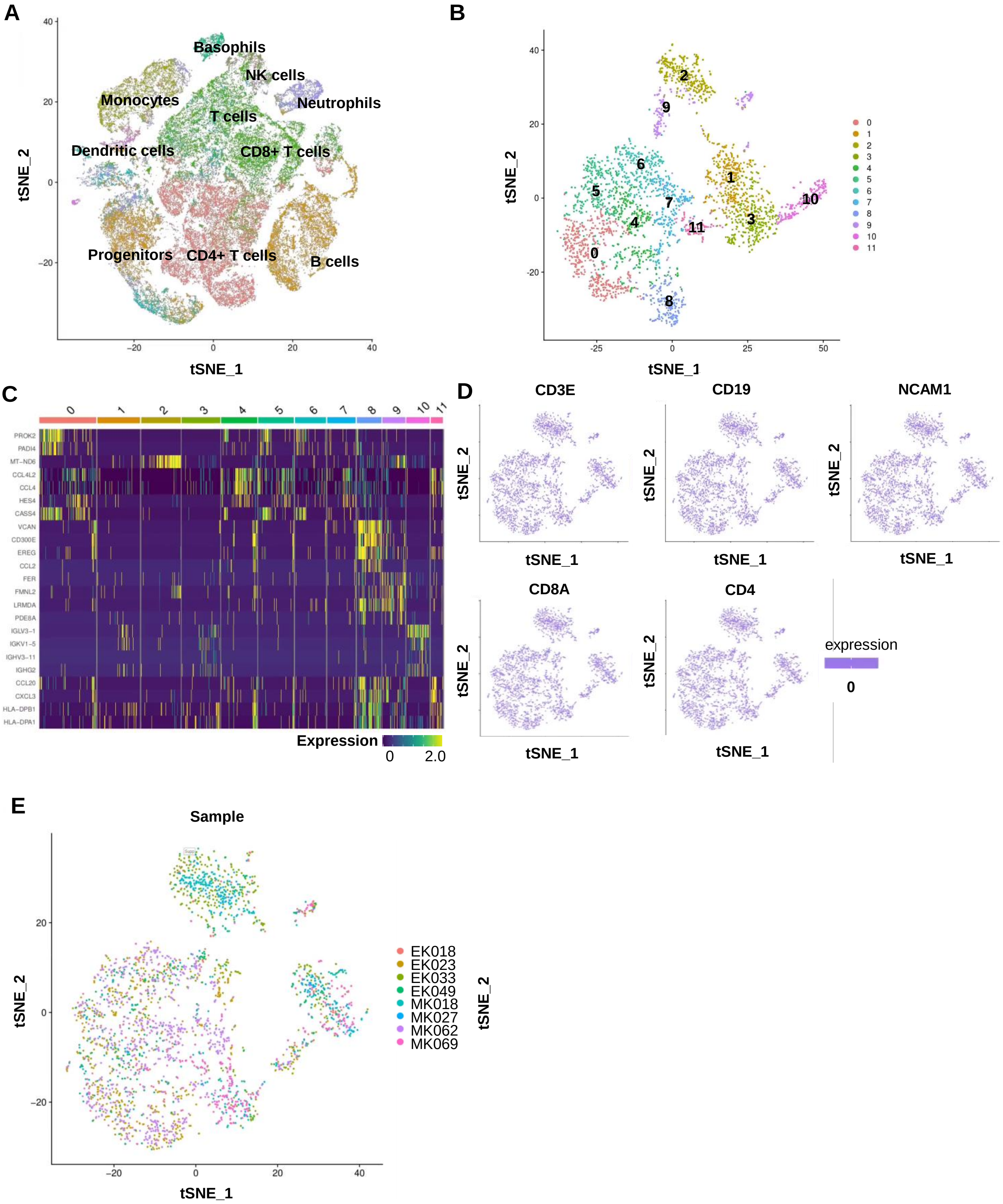

Supplementary Figure 9

Supplementary Figure 9: Single-cell RNA-seq analysis of immune cells from GC and ESCC tissues.

(A) t-SNE of tumor-infiltrating immune cells (CD45-positive cells) from four GC tissues and four ESCC tissues. Cell annotation was performed using SingleR with the Monaco reference. (B) t-SNE of the extracted neutrophil fraction before filtering. (C) Heatmap showing the expression of the top five genes in the neutrophil fraction before filtering. (D) FeaturePlots of neutrophils after filtering, demonstrating the absence of lineage marker gene expression. (E) t-SNE of neutrophils after filtering, color-coded based on anonymized patient IDs.

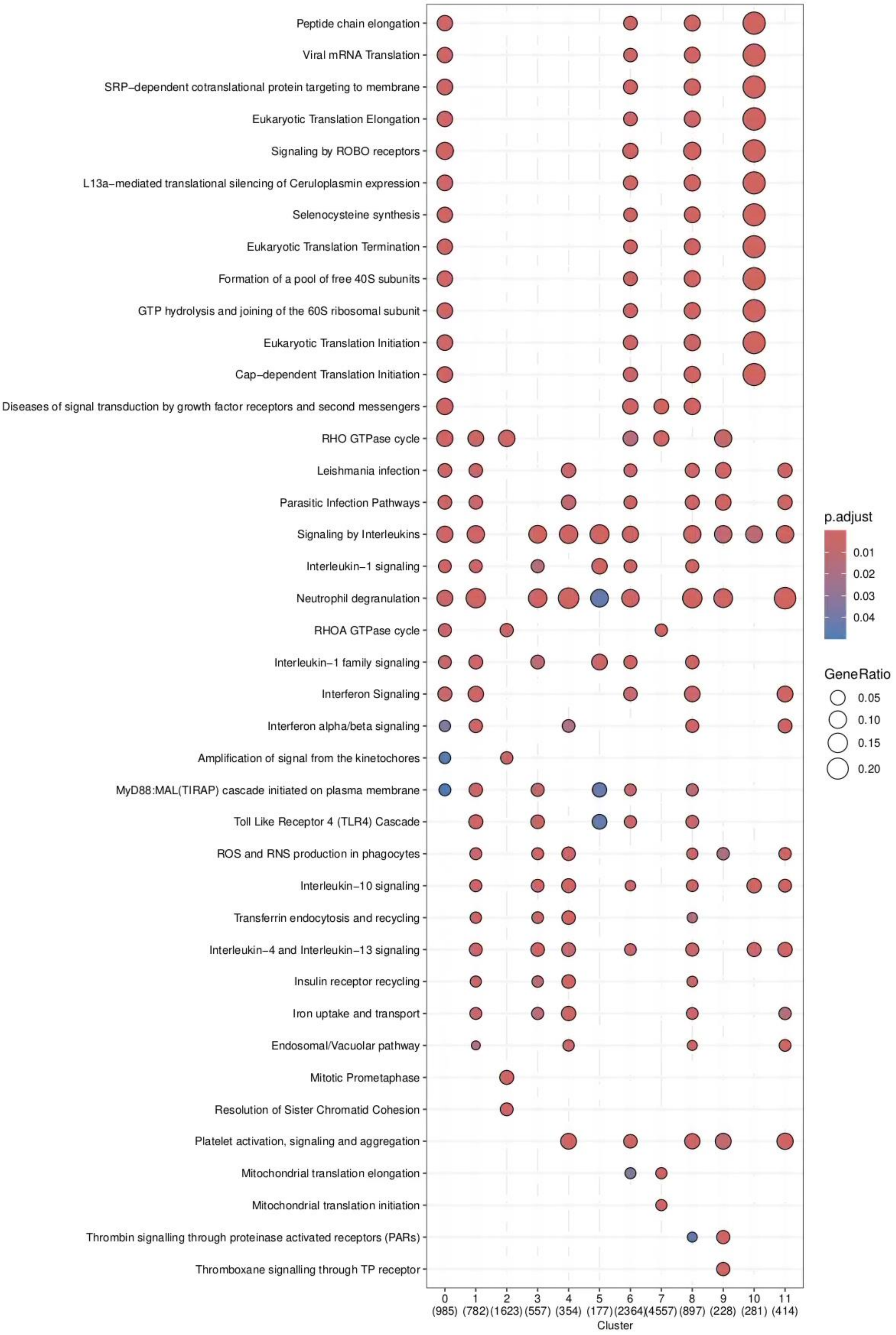

Supplementary Figure 10: Pathway analysis of neutrophil sc-RNAseq data of GC and ESCC tissues. Dot plot of pathway analysis using enrichment analysis with ReactomePA.

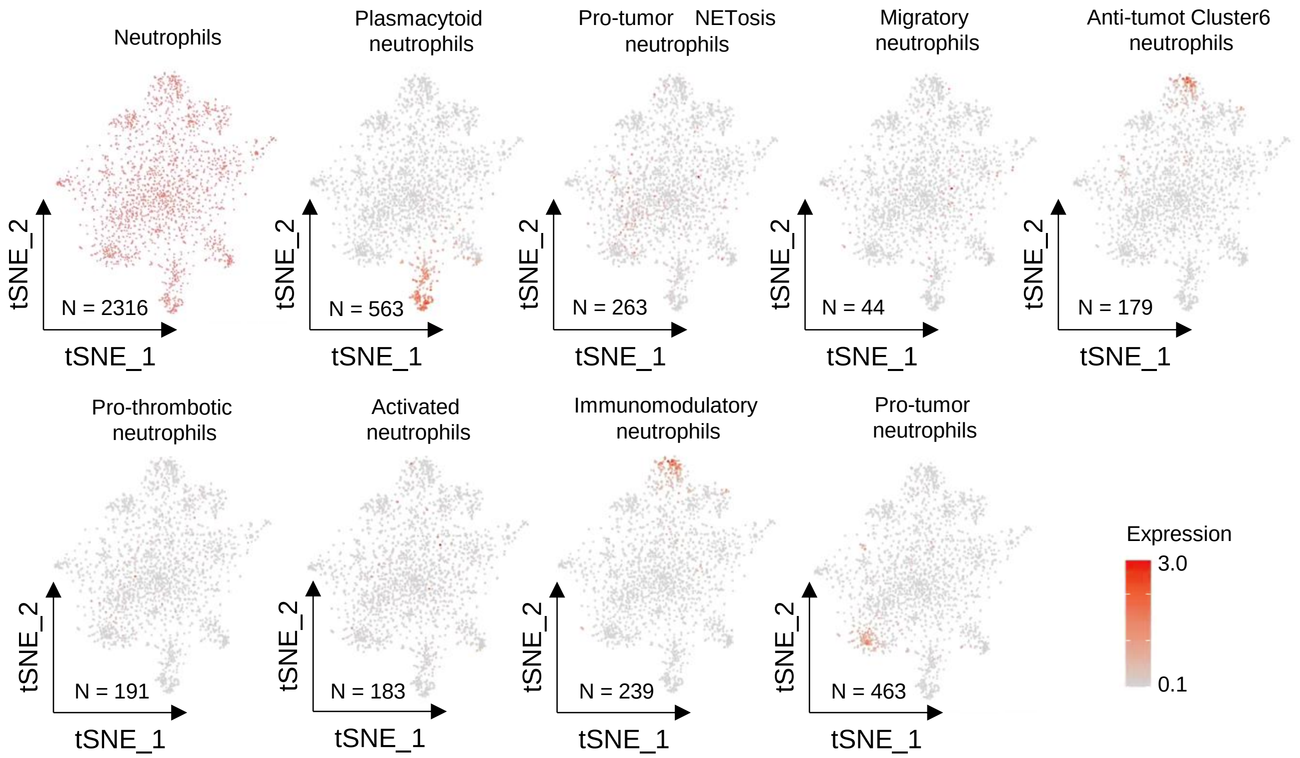

Supplementary Figure 11: Neutrophil presence in stomach tissues in spatial transcriptome (ST) data.

In the spatial transcriptome (ST) data of human gastric mucosa [ref. 26], the presences of specific types of neutrophils were checked. 2,316 neutrophil fractions were extracted and the signatures for each of the neutrophil clusters were checked. The t-SNE plots confirmed the presence of each neutrophil subcluster identified in Figure 4.

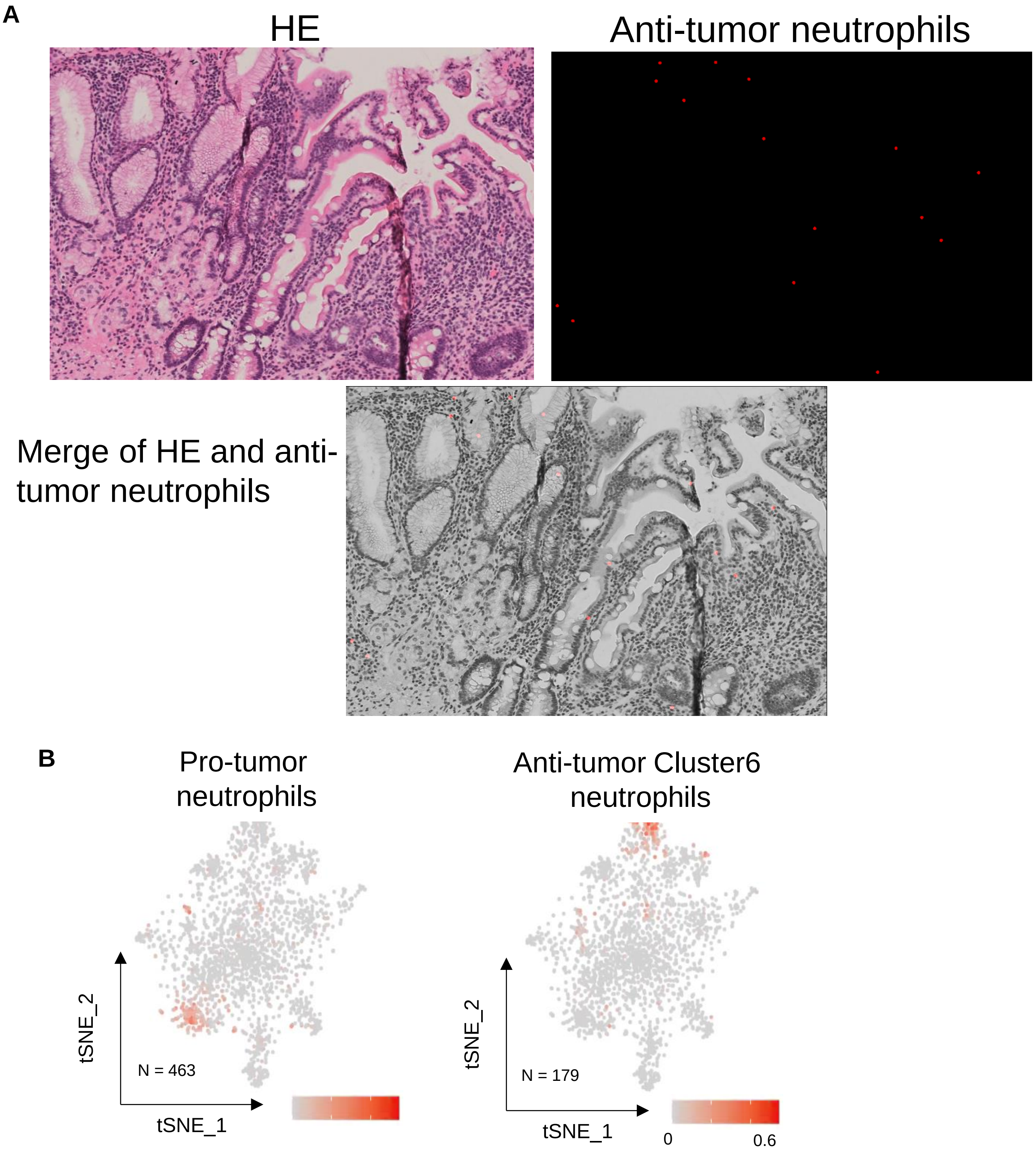

Supplementary Figure 12: Anti-tumorigenic neutrophil signatures in spatial transcriptome data of gastric mucosa.

(A) Identification of neutrophils in ST data from Run5458_5006 [ref.26]. Left: HE stained image, Right: Anti-tumor neutrophils, Bottom: Composite image of HE stained and Anti-tumor neutrophils cells. (B) Feature plots depicting cells expressing Pro-tumor neutrophil (left) and Anti-tumor cluster 6 neutrophil (right) signatures, in t-SNE of neutrophils extracted from ST data of Run5458_5006 and RUN5458_0507 [ref.26].

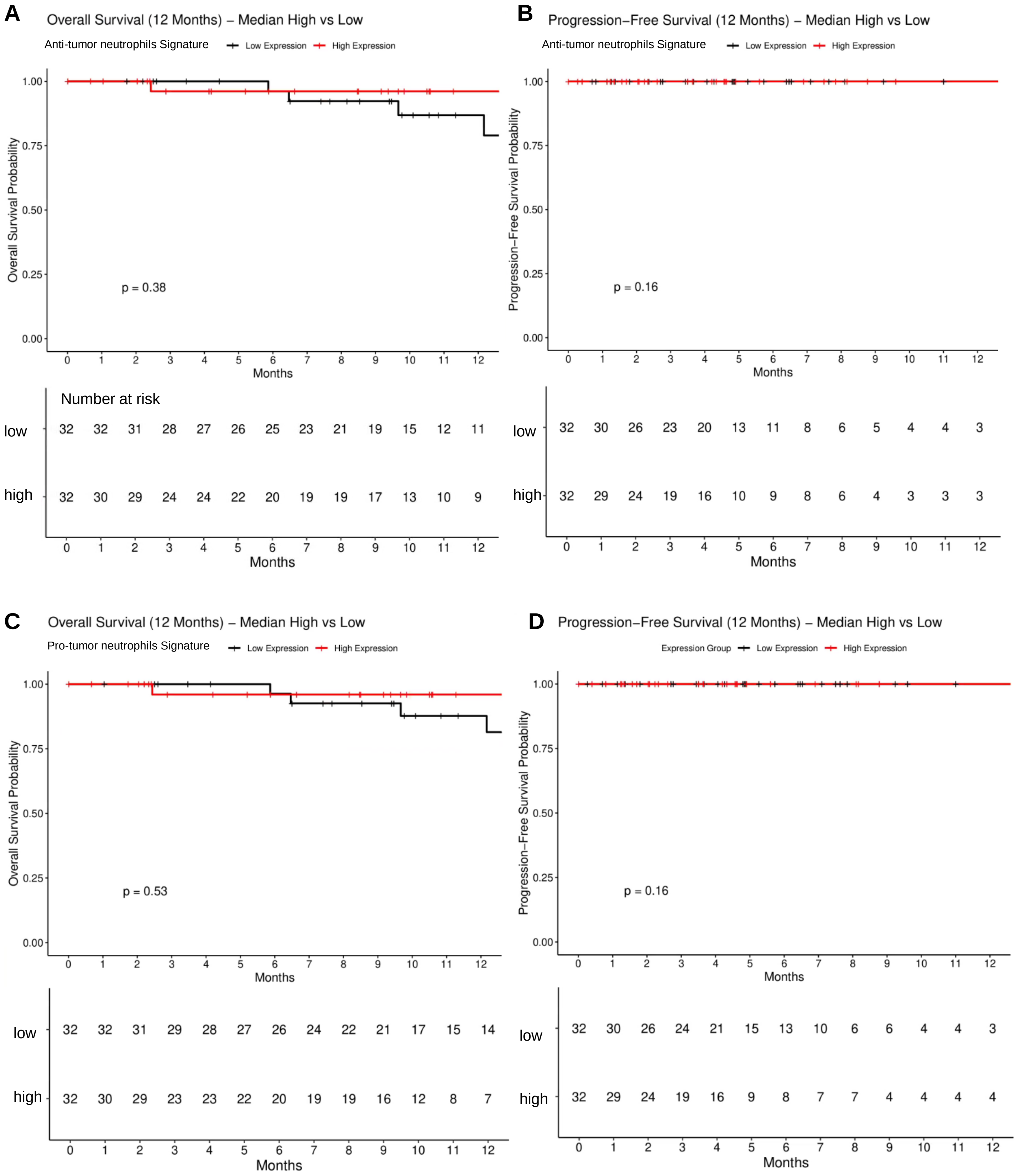

Supplementary Figure 13: Kaplan-Meier plots for classification of Neutrophils signature in GC tissues.

Comparison of survival curves(A) and PFS(B) between groups with high and low Anti-Tumor Neutrophil signatures. Comparison of survival curves(C) and PFS(D) between groups with high and low Pro-Tumor Neutrophil signatures.
